# Multi-round Recycling of Green Waste for the Production of Silver Nanoparticles: Synthesis, Characterization, and Biological Activity

**DOI:** 10.1021/acsomega.5c02607

**Published:** 2025-08-04

**Authors:** Andrea Rónavári, Edi Kachal, Nóra Igaz, Bettina Szerencsés, Bence Kutus, Ilona Pfeiffer, Mónika Kiricsi, Zoltán Kónya

**Affiliations:** † Department of Applied and Environmental Chemistry, Faculty of Science and Informatics, 37442 University of Szeged, Rerrich Béla tér 1., Csongrád-Csanád County, H-6720 Szeged, Hungary; ‡ Department of Biochemistry and Molecular Biology, Faculty of Science and Informatics, University of Szeged, Közép fasor 52., Csongrád-Csanád County, H-6726 Szeged, Hungary; § Department of Biotechnology and Microbiology, Faculty of Science and Informatics, University of Szeged, Közép fasor 52., Csongrád-Csanád County, H-6726 Szeged, Hungary; ∥ Department of Molecular and Analytical Chemistry, Faculty of Science and Informatics, University of Szeged, Dóm tér 7., Csongrád-Csanád County, H-6720 Szeged, Hungary

## Abstract

Due to the limitations of conventional synthesis and the growing utilization of nanoparticles, recent efforts have shifted to green approaches such as utilizing plant waste extracts. A novel initiative to repurpose household and agricultural green waste for the generation of silver nanoparticles (AgNP) offers a sustainable, low-cost alternative that addresses environmental and economic concerns. In this context, we aimed to evaluate the multi-round recyclability of *Coffea arabica* (CA), green tea (GT), and Virginia creeper (VC) waste for AgNP production, then conduct a comprehensive physicochemical and bioactivity characterization of the nanoparticles. The study was designed to prepare AgNPs using waste from CA, GT, and VC generated by one, two, and three rounds of leftover extractions, then the obtained nanomaterials were characterized by transmission electron microscopy (TEM), ultraviolet–visible (UV–vis) spectroscopy, dynamic light scattering (DLS), and X-ray powder diffraction (XRD). Their toxicity on malignant and nonmalignant human cells was evaluated by viability assays, the antimicrobial performance was assessed on Gram-positive, Gram-negative bacteria and against *Cryptococcus neoformans* and *Aspergillus niger* by microdilution method. The production of reactive oxygen species (ROS) was examined by a staining method, and the AgNP-related silver ion release was measured by inductively coupled plasma mass spectroscopy. Our findings confirmed successful synthesis of AgNPs utilizing recycled waste materials; nevertheless, the plant type and extraction round influenced AgNP properties to a unique combination of nanoparticle size and stability. All AgNPs showed strong toxicity against human cancer cells, albeit affecting also noncancerous fibroblasts. GT-derived AgNPs exerted potent antibacterial activity, while those by VC had strong antifungal effects. The observed bioactivity correlated with the increasing number of extraction cycles and was the result of enhanced silver ion-releasing capability that culminated in increased ROS levels. These findings demonstrate the viability of multi-round extract recycling for sustainable AgNP synthesis and suggest potential applications in industrial fields such as antimicrobial air filtration systems.

## Graphical abstract

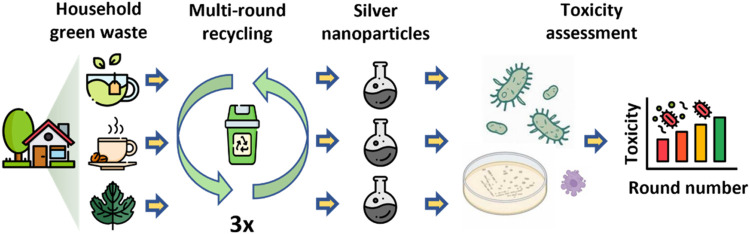

## 1 Introduction

In recent years, inorganic metal and metal oxide nanoparticles, such as gold, silver, and iron oxide, have gained significant attention for their use in electronics, optics, household items, and catalysis, as well as in a broad range of biological applications.^1,2^ Among these, silver nanoparticles (AgNPs) have emerged as particularly valuable materials owing to their tunable physicochemical properties — such as shape, size, surface area, and optical characteristics—which enable a plethora of applications in diagnostics, imaging, drug delivery, and other biomedical fields. Furthermore, AgNPs were shown to have a rather exciting potential to be used effectively in antimicrobial and anticancer treatment modalities.^3–5^ They exhibit their biological action by disrupting cell membranes, generating different types of reactive oxygen species, and inhibiting enzymes, to name a few. AgNPs manifested a broad spectrum of antimicrobial properties, effectively inhibiting the growth of various Gram-positive, Gram-negative bacteria and fungi, including *Streptococcus sanguis*, *Aspergillus fumigatus*, *Candida tropicalis*, multidrug-resistant *Pseudomonas aeruginosa*, and ampicillin-resistant *Escherichia coli* (*E. coli*).^6,7^ Due to AgNPs’ tunable physicochemical properties, dose- and size-dependent biological activity against microbes could be achieved.^8,9^ This has attracted significant interest and guided nanoparticle-based advancements for a wide range of antimicrobial applications.

Although several physical and chemical approaches, such as electrochemical, sono-decomposition, and UV-initiated reductions, have been employed for synthesizing AgNPs, these methods are expensive and use toxic materials, making them less attractive for sustainable utilization.^4^ Green synthesis can be offered as a straightforward, cost-effective, and ecologically friendly technique of production while minimizing waste accumulation.^10–13^ Among the biogenic routes, plant-based synthesis of AgNPs stands out because of its simplicity, efficiency, low cost, and rapid reactions.^14,15^ Plant extracts contain plenty of bioactive agents, such as polyphenols, alkaloids, steroids, tannins, flavonoids, and polysaccharides that serve as reducing or stabilizing agents, yielding metal nanoparticles (NPs) with desirable properties for diverse applications.^11,16,17^

Drawing upon these facts, we published studies on a sustainable and scalable method for synthesizing NPs of iron, gold, and silver, using extracts from *Coffea arabica* (CA), green tea (GT), and Virginia creeper (VC).^17–19^ We assessed the performance of these green NPs in biological systems by comparing them with their chemical counterparts. The findings showed that green NPs could be great alternatives to conventionally produced nanoparticles. It was also demonstrated that green synthesis approaches could offer new frontiers to preserve AgNP toxicity by enhancing colloidal stability.^20^ An even more beneficial and environment-conscious approach could be leveraging the plant waste instead of utilizing fresh plant extracts for nanoparticle production. Agricultural byproducts and household plant wastes, such as coffee grounds, tea bags, fruit and vegetable remains accumulate globally, and their management is not fully resolved. These materials could be repurposed for metal nanoparticle production, offering dual ecological and practical advantages by integrating waste management with the production of green NPs. These green methods not only transform large quantities of waste materials into valuable biocompatible products like NPs but also facilitate their immediate application. Beyond their biological activity, these green-synthesized AgNPs have promising potential for applications in antimicrobial technologies, such as air purification systems and antimicrobial surfaces. When incorporated into air conditioning filters or surfaces, the sustained release of silver ions from AgNPs can inhibit microbial growth, offering an effective solution for improving air quality and hygiene.

On these grounds, our objective was to delineate whether green waste materials, such as GT, CA, and VC, the extracts of which were used previously to produce NPs, could retain their reducing power and be recycled multiple times and utilized in additional synthesis rounds to generate AgNPs. Moreover, we wanted to delineate the physicochemical properties, including size, stability, and morphology of the generated AgNPs and assess their biological activity. According to these objectives, we conducted several rounds of NP syntheses utilizing multiple times recycled residues from GT, CA, and VC, subsequently establishing the physicochemical characteristics of the resultant green AgNPs, followed by an evaluation of their cytotoxic effects on Gram-positive and Gram-negative bacteria, fungi, and both cancerous and noncancerous human cells.

Through this comprehensive methodology, we aimed to ascertain the feasibility of these green NPs derived from recycled plant waste for prospective, scalable, environmentally friendly applications, thereby highlighting their potential in sustainable nanotechnology.

## 2 Materials and Methods

### 2.1 Preparation of Plant Extracts in Multiple Rounds

The selected plant extracts were produced by the method previously reported by Rónavári et al. with some modifications.^17,19^ Virginia creeper (VC, *Parthenocissus quinquefolia*) leaves were collected locally (Szeged, Hungary) in April 2023. The leaves were washed with deionized water to remove any surface dust and dried at room temperature until they achieved constant weight. Afterward, the VC leaves were cut into small pieces. For the first-round extraction, the extracts were produced by using 10 g of VC leaves in 200 mL of deionized water and setting the temperature to 70 °C for 30 min. To remove the impurities, the extracts were filtered (using nylon membrane filters, Whatman 0.45 μM) and then stored at 4 °C until further use. A similar procedure was applied for coffee (CA) and green tea (GT) extracts, except that purchased dry tea leaves (Twinings of London, Green Tea and Lemon) and powdered Turkish coffee (Hazer Baba, *C. arabica*) were boiled directly without any pretreatment. After the first-round extractions, the remaining plant materials (used upon the first extraction, and left behind as “waste”) were dried and recycled two additional times to gain individual extracts each time (second- and third-round extractions) by the same method as described for the first round. The extracts obtained were labeled according to the name of the plant extract and the order number of the extraction round, namely CA1, CA2, and CA3 for coffee, VC1, VC2, and VC3 for Virginia creeper, and GT1, GT2, and GT3 for green tea, respectively. These extracts were stored at 4 °C and then applied individually for AgNP synthesis.

### 2.2 Synthesis of AgNPs

For synthesizing the CA-Ag, VC-Ag, and GT-Ag green AgNPs, the corresponding extracts (of the first-, second-, and third-round extractions) were mixed with 1% aqueous silver nitrate (AgNO_3_, ≥99.0%; Merck, Germany) solution 1:1 volume ratio at 70 °C, pH 7, by continuous stirring for 24 h. Then the prepared nanoparticles were washed and kept at 4 °C for later use.^17,20^ In addition to the green NP synthesis, another sample of AgNPs was also produced by a conventional seed-mediated growth approach.^21,22^ In this method, silver nitrate was reduced by sodium borohydride (SB, NaBH_4_, ≥98.0%; Merck, Germany) to form chemically synthesized silver nanoparticles, referred to as SB-Ag (sodium borohydride-mediated AgNPs). These control AgNPs were compared with the green-synthesized AgNPs in terms of their properties and activities. All syntheses and biological tests were performed in triplicate using standardized conditions to assess reproducibility across batches.

### 2.3 Characterization of Nanoparticles

The morphological properties of nanoparticles, such as the average size, and shape, were assessed by transmission electron microscopy (TEM) with a FEI Tecnai G2 20 X-Twin instrument using 200 kV accelerating voltage (FEI Corporate Headquarters, Hillsboro, OR, calibrated using standard copper grid scale). The size distribution of the samples was determined by evaluating 5 representative TEM images employing the ImageJ software, and histograms were created by using the Origin program 2021 (OriginLab, Northampton, MA). The hydrodynamic size distribution of the samples was measured by dynamic light scattering (DLS) analysis at room temperature using a Malvern Zetasizer Nano ZS instrument (Malvern Instruments, Malvern, U.K., calibrated with 100 nm latex bead standard) with disposable folded capillary cells. The ultraviolet-visible (UV–vis) spectral analysis was recorded to ensure the formation of nanoparticles. The absorbance measurements were carried out over the wavelength range of 300**-**800 nm on an Ocean Optics 355 DH-2000-BAL UV-vis spectrophotometer (calibration performed with standard solution) with 1 cm path**-**length quartz cuvettes. The crystal structures were analyzed by X-ray powder diffraction (XRD). The scans were performed with a Rigaku MiniFlex II powder diffractometer using Co Kα radiation (λ = 1.78897 Å). A scanning rate of 4° min^–1^ in the 20–80° 2θ range was used.

### 2.4 Anticancer Activity

The effect of AgNPs on the viability of cancerous and noncancerous human cell lines was assessed by 3-(4,5-dimethylthiazol-2-yl)-2,5-diphenyltetrazolium bromide (MTT) assays performed on A549 (lung adenocarcinoma), MCF-7 (breast cancer), MCF-7 KCR (multidrug-resistant breast cancer), and MDA-MB-231 (triple-negative breast cancer) tumor cell lines, and MRC-5 noncancerous fibroblast cells. MCF-7 KCR cells are a P-glycoprotein overexpressing multidrug-resistant cell line derived from MCF-7 cells. The cells were maintained in Eagle’s Minimum Essential Medium (EMEM) media (Lonza, Basel, Switzerland) complemented with 10% fetal bovine serum (Biosera, Cholet, France), 2 mM glutamine (Biowest, Nuaille, France), 0.01% streptomycin, and 0.005% penicillin (Biosera, Cholet, France). For this, 1 × 10^4^ cells were seeded into 96-well plates and left to grow. On the following day, cells were exposed to increasing concentrations of AgNPs (0, 5, 10, 20, 40, and 80 ppm, respectively) or equivalent concentrations of AgNP-free extracts. After 24 h incubation, the cells were washed with phosphate-buffered saline (PBS, pH 7.4, Sigma-Aldrich, St Louis, MO), and 5 mg/mL MTT reagent (≥98.0%, Sigma-Aldrich, St Louis, MO) diluted in full culture medium was added to the samples. After 1 h, the MTT reagent was removed, the cells were washed with PBS, and the formazan crystals were dissolved in DMSO (≥99.9%, Sigma-Aldrich, St Louis, MO). The absorbance of the samples was determined at 570 nm (background at 630 nm) using a Synergy HTX plate reader (BioTek, Biotek Instruments Inc., Winooski, VT, wavelength calibration performed with standard solution).

The cytotoxicity of AgNPs on A549 tumor cells was investigated further using the lactate dehydrogenase (LDH) assay. For this, tumor cells were seeded into 96-well plates at 1 × 10^4^ density. On the following day, the cells were treated with 20 ppm of AgNPs or with equivalent amounts of AgNP-free extracts. In the positive control, the cells were exposed to 0.1% Triton-X-100 detergent (≥98.0%, Sigma-Aldrich, St Louis, MO). After a 24 h treatment, the supernatant of the cells was transferred to a new 96-well plate, and an LDH reaction mixture (Sigma-Aldrich, St Louis, MO) was added to the supernatant. After 1 h of incubation, the absorbance of the samples was measured at 490 nm using a Synergy HTX plate reader (BioTek, Biotek Instruments Inc., Winooski, VT).

### 2.5 Antimicrobial Activity

The inhibitory effects of nanoparticles on the growth of *E. coli* SZMC 0582 (SZMC: Szeged Microbiology Collection), *Bacillus megaterium* (*B. megaterium*) SZMC 6031, *Cryptococcus neoformans* (*C. neoformans*) IFM 5844 (IFM: Research Center for Pathogenic Fungi and Microbial Toxicoses, Chiba University), and *Aspergillus niger* (*A. niger*) SZMC 0050 strains were assessed in 96-well microtiter plates. The minimal inhibitory concentration (MIC) was established through the microdilution technique, where 50 μL of a serially diluted nanoparticle solution was combined with 50 μL of cell suspension (8 × 10^4^ cells/mL) in RPMI 1640 medium (Biosera, Cholet, France). For inoculation of *A. niger*, conidium suspension was used (2 × 10^4^ conidia/ml in RPMI 1640 medium). The concentration spectrum in which each nanoparticle was applied ranged from 80 to 0.15 ppm, and the dilutions were prepared with RPMI 1640 medium. Following a 48 or 72 h incubation period at 30 °C, the optical density of the cultures was assessed at 620 nm using a SPECTROstar Nano plate reader (BMG LabTech, Offenburg, Germany, wavelength calibration performed with standard solution). Bacteria were cultivated for 48 h while *C. neoformans* and *A. niger* for 72 h. A cell suspension supplemented with 50 μL of RPMI 1640 served as the growth control. The minimal inhibitory concentration was defined as the threshold for growth inhibition of ≥90% in comparison to the 100% growth observed in the untreated control. The experiments were carried out in three biological repeats, always in triplicate.

For the assessment of cell viability, 5 μL from each well was transferred onto solid media, and the growth of the strains was evaluated after a 48 h incubation period at 30 °C. Yeast extract-peptone-dextrose (YPD, 1% yeast extract, 2% peptone, 2% dextrose, and 2% agar) medium was utilized for the cultivation of *C. neoformans* and *A. niger*, while *E. coli* and *B. megaterium* were propagated on meat extract-peptone medium (1% dextrose, 0.5% meat extract, 0.5% peptone, 0.1% yeast extract, and 2% agar). All ingredients of the media were purchased from Sigma-Aldrich, St Louis, MO.

### 2.6 Measuring ROS Production

The production of reactive oxygen species (ROS) following treatment with AgNPs (CA1-, CA2-, CA3-, GT1-, GT2-, GT3-, VC1-, VC2-, VC3-AgNP, and SB-Ag) was evaluated on *C. neoformans* utilizing 2’,7’-dichlorofluorescein diacetate (DCFDA) staining. *C. neoformans* cells were inoculated into RPMI 1640 medium at a concentration of 5 × 10^4^ cells/mL, and treated with AgNPs at half-MIC concentration, followed by a 24 h incubation at 30 °C. Cells treated with H_2_O_2_ at a concentration of 0.08% under the same cultivation conditions served as a positive control. Following the treatments, the cells were harvested by centrifugation (5000 *g*, 10 min, room temperature), washed once with PBS, and all the samples were suspended in 0.5 mL RPMI 1640 medium containing 10 μM DCFDA (Sigma-Aldrich, Saint Louis, MO) and incubated in the dark for 1 h at 30 °C. The overall fluorescence intensity of the samples was quantified using flow cytometry (FlowSight, Amnis-EMD Millipore, Burlington, MA). The measurements were conducted in triplicate, utilizing three independent biological replicates.

### 2.7 Inductively Coupled Plasma Mass Spectroscopy (ICP-MS)

The concentration of Ag was quantified by applying an Agilent 7900 ICP-MS spectrometer equipped with an automatic sampler. Samples and calibration series were prepared and diluted appropriately using deionized water (Milli-Q, Merck, Germany). Concentrated nitric acid (for trace analysis, NORMATOM, VWR Chemicals) was added to each sample with a final concentration of ca. 1 wt %. In addition, samples contained 0.1 ppm yttrium (Y; for trace analysis, ARISTAR, VWR Chemicals) used as an internal standard. As for Ag, the intensities of 107Ag and 109Ag isotopes were used, whereas for Y, the intensity of the 89Y isotope was used. Ion counts were converted to concentrations based on an 11-member calibration series with Ag total concentrations ranging from 0 to 1 ppm, prepared from a multielement standard solution (for trace analysis, ARISTAR, VWR Chemicals). Intensities were also measured in helium collision mode.

## 3 Results

Green AgNP synthesis was performed by utilizing various plant materials that were reused in multiple rounds of extraction. During the synthesis reaction, when the aqueous green extracts were added to the silver nitrate-containing reaction mixture, we observed a color change that varied in tint and manner; the conversion was from yellowish to brown or gray. These color changes were indicative of the bioreduction of silver ions to form NPs. As a reference, SB**-**Ag nanoparticles were also prepared by conventional chemical reduction using sodium borohydride instead of the green extracts.

### 3.1 TEM Measurements

Since the morphological characteristics are crucial factors that determine the physicochemical properties of nanoparticles, the shape, particle size, and particle size distribution of the obtained AgNPs were characterized using transmission electron microscopy. Figure 1. illustrates the representative TEM micrographs of nanoparticles produced by green reagents of multi-round extractions as well as by conventional chemical reagents. According to TEM images, in each of the syntheses, samples exhibited isotropic morphology. Upon the first round of extracts when CA1, VC1, and GT1 were used, the Ag nanoparticles were well-formed with only minor polydispersity, but no aggregation was observed (Figure 1). When the CA1 extract was used as a reductant and capping agent, the particle sizes were slightly larger (20.9 ± 6.7 nm) compared to VC1 (13.6 ± 3.9 nm) and GT1 (9.2 ± 3.6 nm) mediated particles. For nanoparticles obtained with the extracts of the second and third extraction round of coffee waste, i.e., CA2 and CA3, the size of nanoparticles was slightly smaller compared to the NPs obtained with the first round extract, and the average particle size was around 14.8 ± 5.1 and 13.9 ± 4.4 nm respectively, as shown in the histogram (Figure 1). In the case of VC plant waste material, VC1-AgNPs were smaller in size compared to those obtained with second- and third-round extracts, VC2- (22.9 ± 5.7 nm) and VC3-AgNPs (20.0 ± 4.6 nm). Nevertheless, in the case of GT, the synthesis with all the extracts yielded particles of similar size, but these were all significantly smaller than the NPs obtained with the CA or VC respective extracts (the particle size of GT-mediated NPs was around ∼10 nm). Comparatively, AgNPs synthesized with sodium borohydride were around 10 nm.

**Figure 1:**
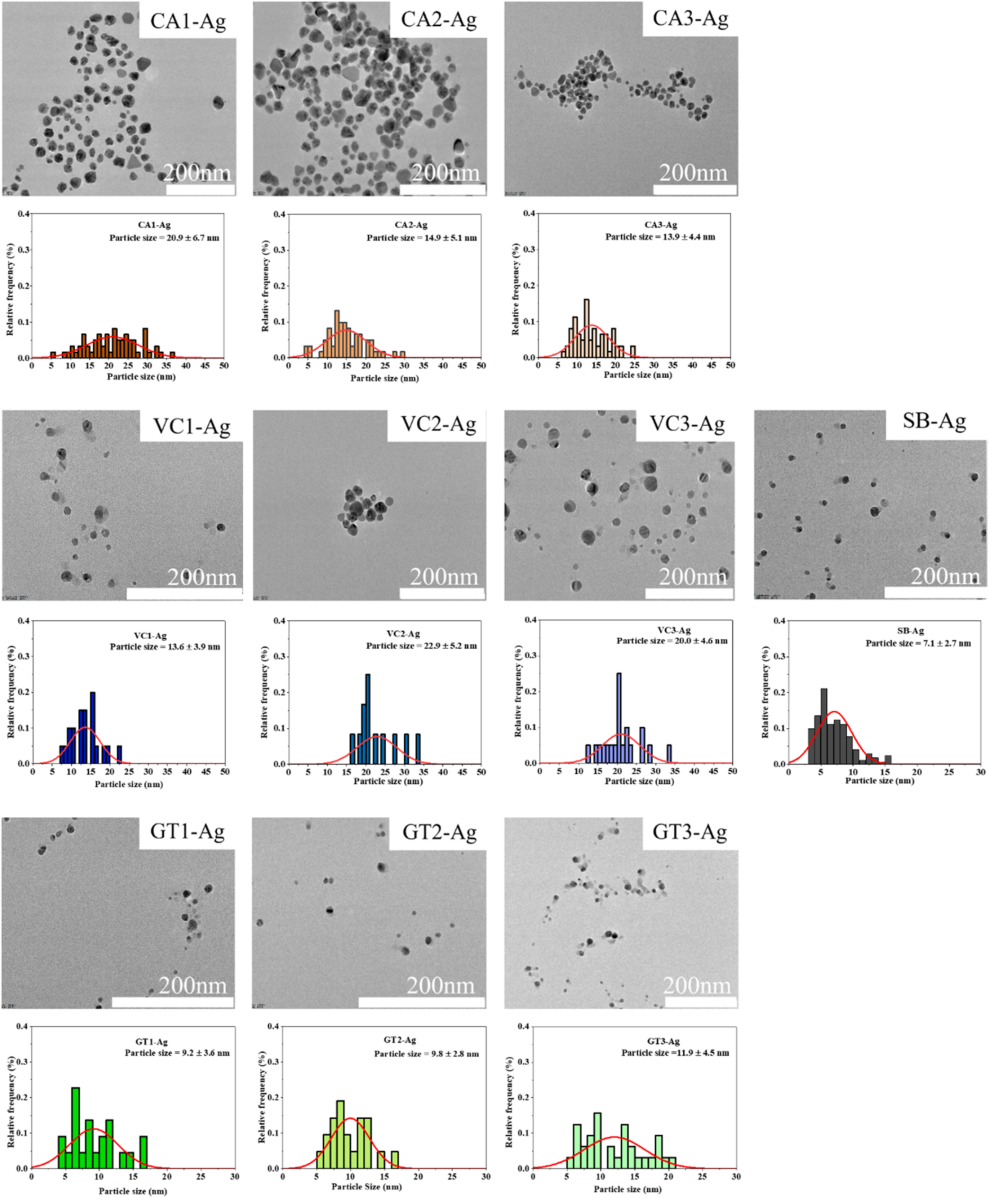
Morphology, size and size distributions of AgNPs produced by using various green waste extracts obtained in three extraction rounds of coffee (CA1-Ag, CA2-Ag, CA3-Ag), Virginia creeper (VC1**-**Ag, VC2-Ag, and VC3-Ag), green tea (GT1-Ag, GT2-Ag, and GT3-Ag), and by chemical method using sodium borohydride (SB-Ag) obtained by TEM and TEM image analyses.

### 3.2 DLS Measurements

DLS measurements were carried out to determine the average hydrodynamic diameter and the ζ-otential of the AgNP samples. These data are shown in Figure 2. together with the average particle sizes obtained by TEM image analyses for comparison purposes. The largest hydrodynamic diameters of the coffee-mediated AgNP samples belonged to those particles that had been prepared with CA1, since AgNPs obtained with the second and third rounds of CA extracts had smaller diameters (Figure 2). This tendency in the differences of hydrodynamic diameters of CA-extract generated nanoparticles (CA1-Ag > CA2-Ag > CA3-Ag) was also observed for the size distribution of the AgNPs measured by TEM image analysis (Figures 1 and 2).

**Figure 2:**
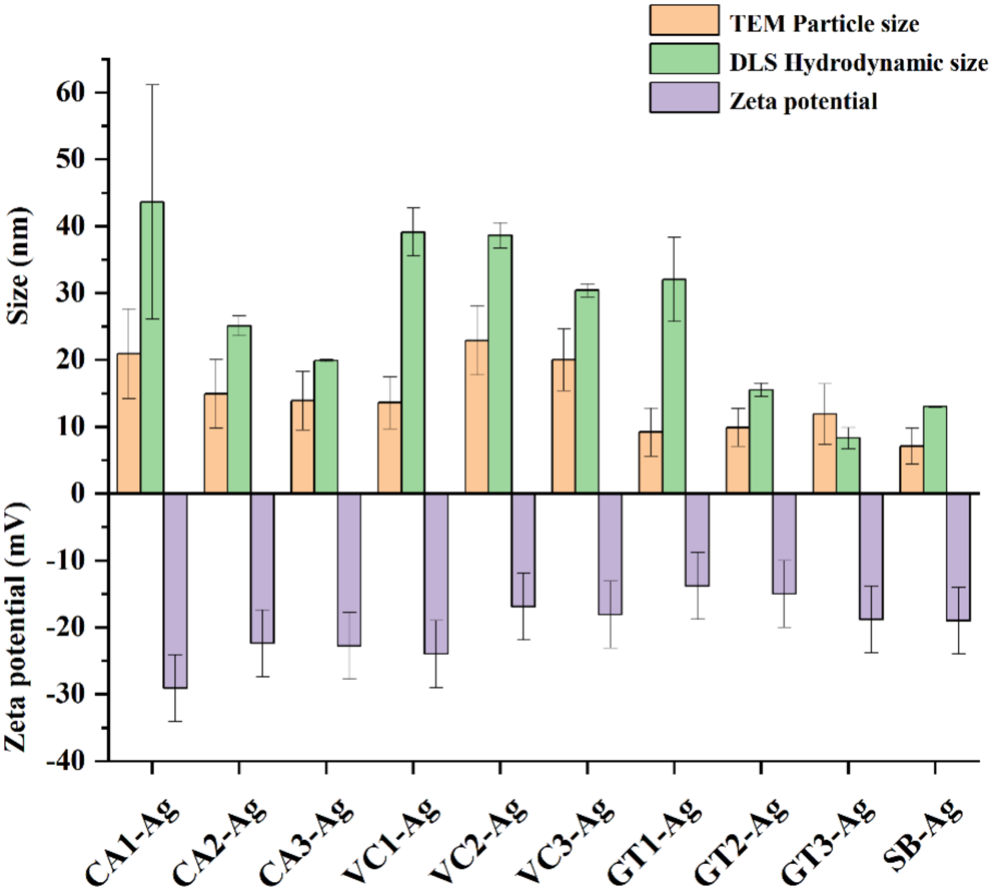
Average particle size obtained by TEM image analyses (orange), the average hydrodynamic size (green), and ζ-potential (purple) values assessed by DLS of AgNPs produced with various green waste extracts of three extraction rounds. Abbreviations stand for AgNPs generated by 1st, 2nd, and 3rd extracts of coffee: CA1-Ag, CA2-Ag, CA3-Ag, Virginia creeper: VC1-Ag, VC2-Ag, and VC3-Ag, green tea: GT1-Ag, GT2-Ag, and GT3-Ag, and in a chemical method using sodium borohydride: SB-Ag. Error bars indicate mean ± standard deviation.

Moreover, ζ-potential values showed that CA2-Ag and CA3-Ag samples were less stable (−22.4 mV; −22.8 mV) compared to the first round AgNPs (CA1-Ag: −29.1 mV). A similar decreasing tendency in hydrodynamic diameter and stability could be observed in the case of VC samples, namely, smaller hydrodynamic diameter with less negative ζ-potential values were measured for third round green extract-assisted AgNPs than for first extract-produced AgNPs (VC1-Ag). Interestingly, although the hydrodynamic diameter of GT extract-assisted particles decreased by extraction rounds (GT1-Ag > GT2-Ag > GT3-Ag), the particle sizes measured by TEM did not follow this tendency, and the ζ-potential values also indicated greater stability for GT3-Ag compared to GT1-Ag (Figure 2).

### 3.3 X-ray Powder Diffraction (XRD) Measurements

The crystalline structure and phase purity of AgNPs synthesized chemically and via the green multi-round extraction method were systematically analyzed through X-ray diffraction (XRD) (Figure 3). In all samples, the characteristic diffraction peaks corresponded to the (111), (200), (220), and (311) crystal planes of face-centered cubic (FCC) silver (JCPDS card no. 04–078).^16^ These reflections confirmed the successful formation of AgNPs with every extract type, even after successive reuse of plant materials. Notably, XRD analysis was performed using Co Kα radiation (λ = 1.78897 Å), which results in a systematic shift in 2θ values compared to the more commonly used Cu Kα radiation. Peak intensities declined with successive extraction, reflecting decreased phytochemical availability, a trend consistent with phytochemical exhaustion. Some additional peaks appeared in later extraction rounds (especially in CA3-Ag, VC3-Ag, GT3-Ag) in the 25–35° and 55–65° ranges, suggesting possible residual organic material or the presence of secondary silver phases such as Ag_2_O.

**Figure 3:**
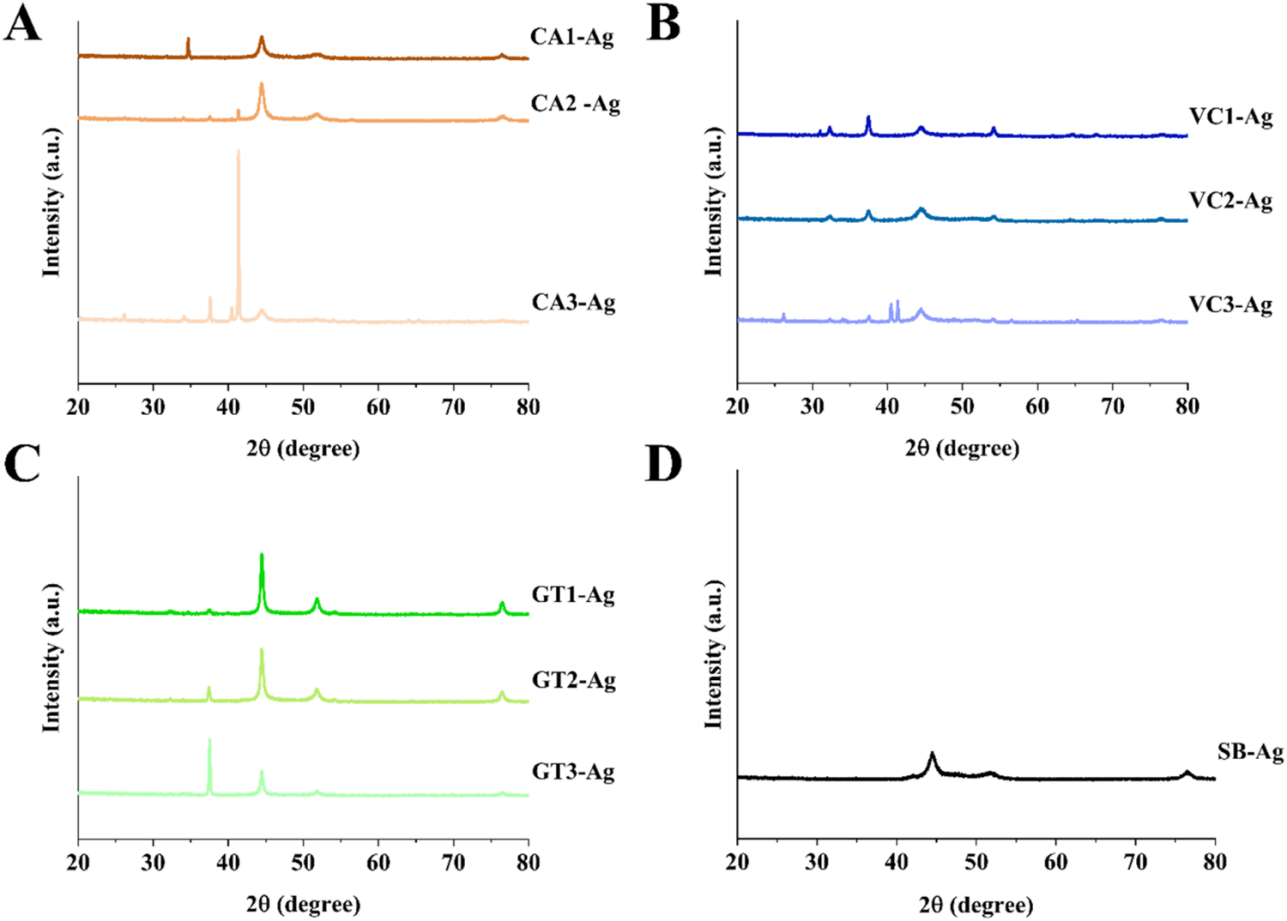
X-ray diffractograms of AgNPs produced by using various green waste extracts obtained in three extraction rounds of coffee (CA1-Ag, CA2-Ag, CA3-Ag) (A), Virginia creeper (VC1-Ag, VC2-Ag, and VC3-Ag) (B), green tea (GT1-Ag, GT2-Ag, and GT3-Ag) (C), and by chemical method using sodium borohydride (SB-Ag) (D).

### 3.4 UV–visible Spectral Analysis

The reduction of silver ions to the nanoparticle form was monitored by measuring the UV–visible spectra of the green extract-mediated process as well as the classic chemical reaction using sodium borohydride. The absorbance spectrum with a spectral maximum in the 400 to 450 nm region is characteristic to surface plasmon resonance (SPR) of spherical nanosilver, confirming the presence of AgNPs in the suspension (Figure 4). The maximum of the characteristic absorbance spectra of the coffee-mediated nanosilver suspensions was at 449.99 nm for CA1-Ag, 439.59 nm for CA1-Ag, and 432.76 nm for CA3-Ag, respectively (Figure 4A). The characteristic SPR peaks for VC-mediated samples are observed at 440.36, 441.8, and 431.94 nm for VC1-Ag, VC2**-**Ag, and VC3-Ag, respectively (Figure 4B), while for GT-mediated samples, the peaks are at 459.7 nm for GT1-Ag, 442.21 nm for GT2-Ag, and 440.15 nm for GT3-Ag, respectively (Figure 4C). The UV-vis spectra obtained from AgNP suspensions that were synthesized by chemical reaction showed specific peaks at 400.13 nm (Figure 4D). These absorbance values are indicative of surface plasmon resonance and support the formation of AgNPs.^21,23^

**Figure 4:**
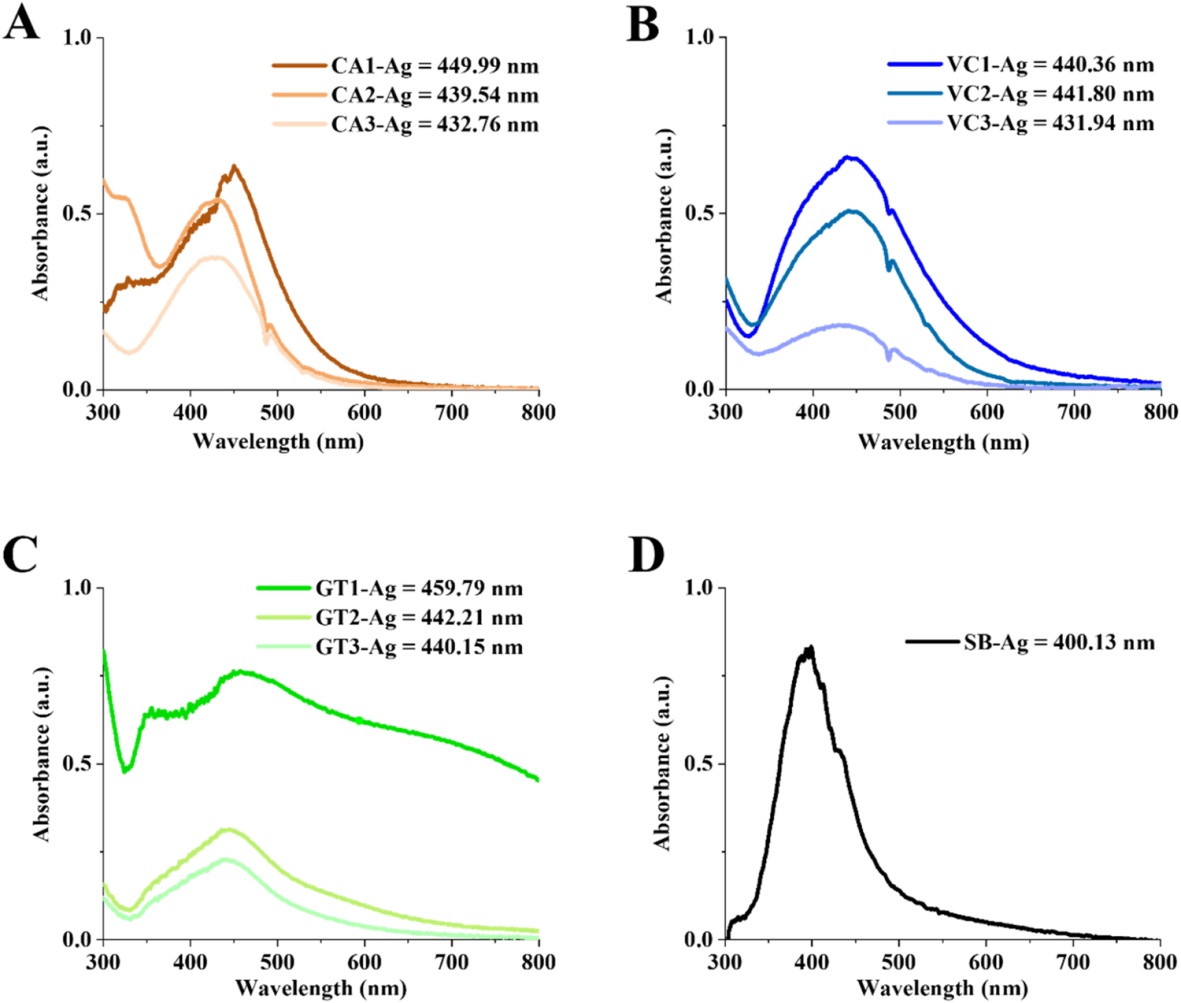
UV–vis absorption spectra of AgNPs produced by using various green waste extracts obtained in three extraction rounds of coffee (CA1-Ag, CA2-Ag, CA3-Ag) (A), Virginia creeper (VC1-Ag, VC2-Ag, and VC3-Ag) (B), green tea (GT1-Ag, GT2-Ag, and GT3-Ag) (C), and by chemical method using sodium borohydride (SB-Ag) (D).

### 3.5 Anticancer Activity

The biological activity of each green extract and every AgNP sample generated by these extracts was tested on human cancerous cell lines (A549, MCF-7, MCF-7 KCR, and MDA-MB-231 cells) and noncancerous fibroblast cells (MRC-5) using MTT and LDH assays. According to the results of the MTT assays performed on the extract-treated cells, only the CA3 and VC2 extracts, and in singular cases, CA2 or VC3 extracts, decreased somewhat the viability of some cell lines, usually at the highest applied concentration. Nevertheless, only slight differences exerted on cell viability were observed between the first-, second-, or third-round extracted CA and VC solutions (Figure S1). Regarding the GT waste extracts, GT1 and GT3 induced slight toxicity in some cell lines; significant differences were observed between the toxicity of GT3, GT2, and GT1 treatments in the higher applied extract concentrations (Figure S1). Interestingly, VC1 and GT2 occasionally enhanced the viability of cells compared to the untreated controls.

The AgNPs produced by CA-, VC-, and GT extracts significantly decreased the viability of all the tested cell lines at various concentrations (Figure 5). The most sensitive cell line was MCF-7, since every CA-, VC-, and GT-AgNP treatment at each applied concentration was highly toxic to these cells. In this case, no observable difference was detected between the first, second, and third round-extract prepared CA-, VC-, or GT-AgNPs. In general, VC-AgNPs were more toxic to the cells than CA-, or GT-AgNPs, since VC-AgNPs were massively toxic already at 5 ppm concentration. In many cell types, we observed differences between the impact of AgNPs prepared with the first, second, and third-round extracts of CA-, VC-, and GT. Remarkably, AgNPs synthesized with the first green extracts (CA1, VC1, GT1) showed lower toxicity compared to the AgNPs generated with second or third-round extracts. Often, third-round green extract-mediated AgNPs showed the highest toxicity on the tested cell lines. On certain cell lines, no difference was observed between the effects of the second and third-round AgNPs. According to the results of the viability measurements, CA-, VC-, and GT-AgNPs exhibit no cancer-selective effect since a similar viability decrease was observed on AgNP-treated MRC-5 fibroblast cells as on the cancerous cell lines (Figure 5).

**Figure 5:**
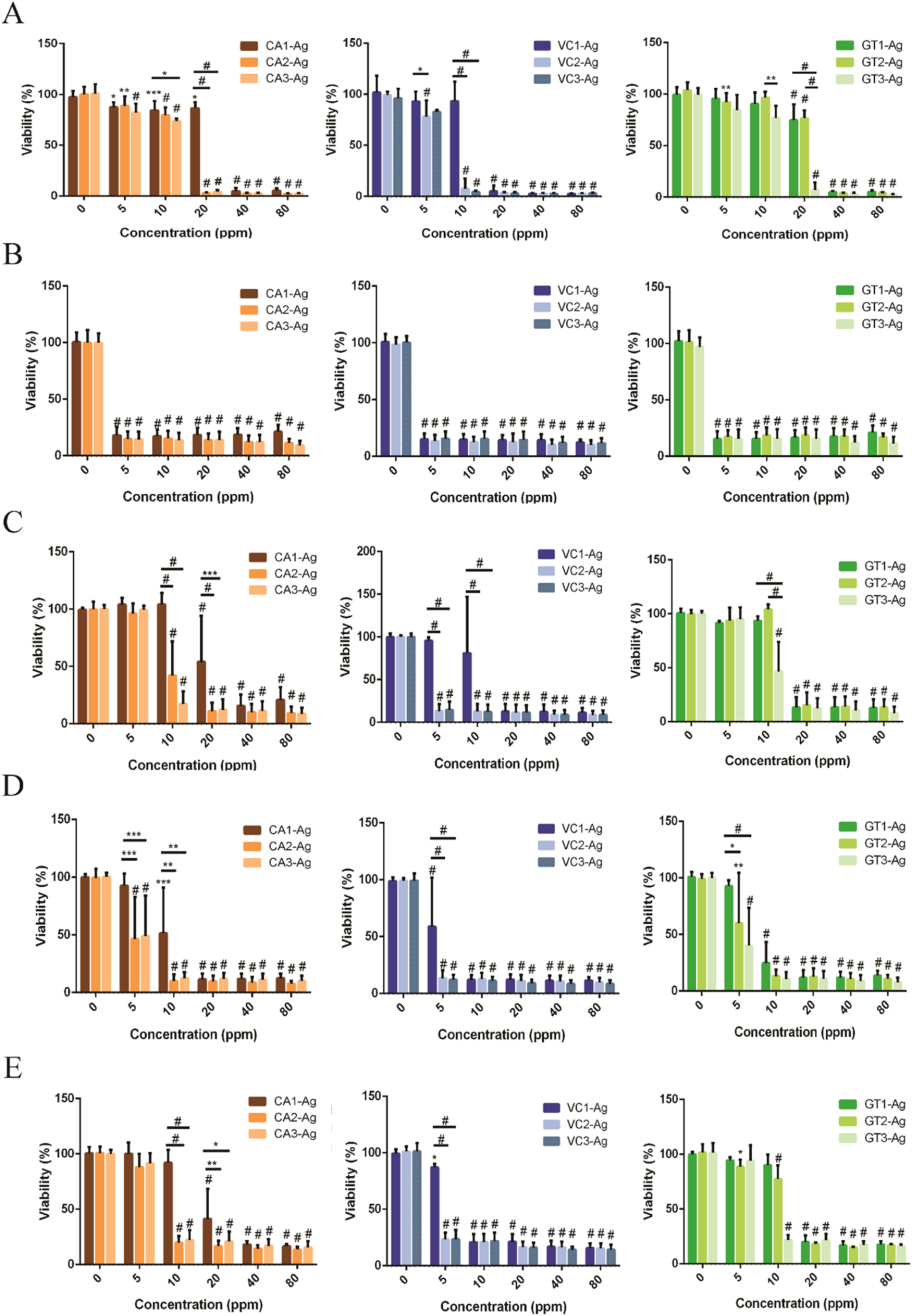
Viability of (A) A549, (B) MCF-7, (C) MCF-7 KCR, (D) MDA-MB-231 cancerous, and (E) MRC-5 noncancerous cells after 24 h of CA-, VC-, and GT-AgNP treatments. Two**-**way ANOVA, Tukey’s multiple comparisons test, *P < 0.05; **P < 0.005; ***P < 0.001; ^#^ *P* < 0.0001.

We wanted to know whether the decrease in cell viability observed upon green AgNP exposures is the result of cell necrosis; therefore, we performed an LDH release assay on A549 cells treated with the green extracts and in parallel experiments on cells exposed to the corresponding green AgNPs. Results of the LDH assay showed no difference in the LDH activity of the supernatant of untreated control cells and the CA**-**, VC-, and GT-AgNP-treated cells or the corresponding extracts, indicating that the treatments do not induce necrosis in the cells (Figure 6).

**Figure 6:**
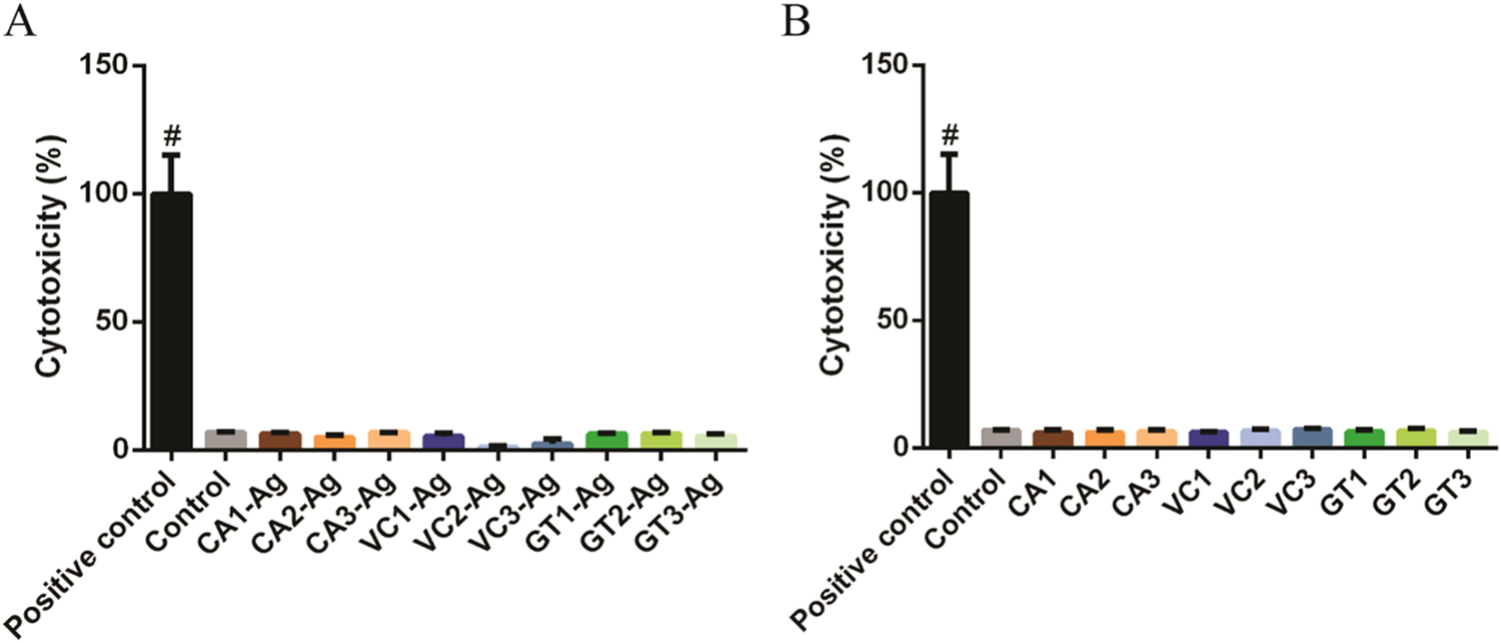
Potential necrosis of A549 cells upon CA-Ag, VC-Ag, or GT-Ag treatment (A) and upon CA, VC, or GT extracts (B) detected by LDH assay. One-way ANOVA, Tukey’s multiple comparisons test, ^#^ *P* < 0.0001.

### 3.6 Antimicrobial Activity

The antibacterial efficacy of the individual extracts (of coffee, green tea, Virginia creeper) as well as of AgNPs synthesized utilizing these extracts (CA-Ag, GT-Ag, VC-Ag) was tested and evaluated against *B. megaterium* and *E. coli*, while *A. niger* and *C. neoformans* were employed to assess the antifungal performance of the extracts and AgNPs. All nanoparticles formulated from green extracts demonstrated both antibacterial and antifungal properties (Table 1), whereas the green waste extracts (CA, GT, VC) applied as reducing agents during the nanoparticle preparation did not exhibit any antimicrobial activity (Table S1). Regarding AgNPs produced by the first coffee extract, among the bacteria and fungi tested, *B. megaterium* exhibited the highest susceptibility (MIC: 5.00 ppm) toward CA1-Ag, whereas *E. coli* displayed lower susceptibility with a MIC of 40.00 ppm. The MIC of CA1-Ag for *A. niger* and *C. neoformans* was determined to be 20 and 10 ppm, respectively (Table 1).

**Table 1:** Minimal Inhibitory Concentration of Multi-Round Extract-Generated Green AgNPs against Bacteria (*E. coli* and *B. megaterium*) and Fungi (*A. niger* and *C. neoformans*)­^a^

	*B. megaterium* SZMC 6031	*E. coli* SZMC 0582	*A. niger* SZMC 0050	*C. neoformans*IFM 5844
CA1-Ag	5.00	40.00	20.00	10.00
CA2-Ag	2.50	20.00	5.00	5.00
CA3-Ag	2.50	10.00	1.25	5.00
GT1-Ag	1.25	80.00	10.00	5.00
GT2-Ag	0.62	80.00	5.00	5.00
GT3-Ag	1.25	20.00	5.00	5.00
VC1-Ag	5.00	80.00	10.00	5.00
VC2-Ag	2.50	40.00	1.25	2.50
VC3-Ag	2.50	20.00	0.62	2.50

a AgNPs were produced by using various green waste extracts obtained in three extraction rounds of coffee (CA1-Ag, CA2-Ag, CA3-Ag), Virginia creeper (VC1-Ag, VC2-Ag, and VC3-Ag), and green tea (GT1-Ag, GT2-Ag, and GT3-Ag).

Notably, subsequent extraction rounds of CA enhanced the activity of the obtained nanoparticles. The MICs were effectively reduced by half or quarter when using CA2-Ag, and decreased even more (to 16th of CA1-Ag for *A. niger*) by applying third extract-based AgNPs (CA3-Ag). Nanoparticles synthesized from Virginia creeper extract (VC1-Ag, VC2-Ag, VC3-Ag) exhibited comparable activity to CA1-Ag, CA2-Ag, and CA3-Ag against *B. megaterium* (with MIC values of 5.00, 2.50, and 2.50, respectively). The observed MIC values of all three VC-Ag for *C. neoformans* were the same as for *B. megaterium*. Conversely, *E. coli* exhibited reduced susceptibility, as MIC values halved each time for AgNPs from subsequent extraction rounds (80.00, 40.00, and 20.00 ppm, respectively). *A. niger* was the most sensitive to VC-Ag nanoparticles since the MIC decreased from 10.00 to 0.62 ppm for VC1-Ag and VC3-Ag, respectively. Among the various green AgNPs, the green tea extract-mediated nanoparticles yielded the most potent particles against *B. megaterium*. The MIC values were recorded at 1.25 ppm for GT1-Ag and GT3-Ag, but were even lower (0.62 ppm) for GT2-Ag. The susceptibility of *E. coli* was much lower than that of *B. megaterium*, as MIC values on *E. coli* for GT1-Ag and GT2-Ag were 80.00 ppm, decreasing to 20.00 ppm for GT3-Ag. Regarding *C. neoformans*, its susceptibility to GT-Ag was the same regardless of the extract used to produce the nanoparticle, with MIC values being consistently 5.00 ppm across all three instances. The MIC of GT1-Ag for *A. niger* was 10.00 ppm, while it was reduced by half for GT2-Ag and GT3-Ag (5.00 ppm in both cases). Interestingly, in many cases, the AgNPs prepared with the first green extract were less toxic to microbes than the nanoparticles generated with recycled, 2- or 3-times-extracted green materials.

The bactericidal or fungicidal effect of the nanoparticles was checked by testing the viability of CA-Ag, GT-Ag, or VC-Ag-treated cells. All the tested AgNPs proved to be bactericidal and fungicidal as well (Figure S2). The bactericidal and fungicidal concentrations required for the complete elimination of the microbial cells were equal to the minimum inhibitory concentrations.

Since AgNPs possess the capacity to elicit biological activity through the generation of reactive oxygen species (ROS), we examined the amount of ROS produced by AgNP treatments in *C. neoformans*. The cells were treated with CA1-Ag, CA2-Ag, CA3-Ag, VC1-Ag, VC2-Ag, VC3-Ag, GT1-Ag, GT2-Ag, and GT3-Ag, respectively, at their half-MIC concentration for 24 h, followed by staining with DCFDA. DCF fluorescence intensity values revealed that AgNPs derived from coffee, green tea, or Virginia creeper waste extracts significantly enhanced ROS production relative to untreated control samples (Figure 7). There was no statistically significant variation in the ROS levels generated by AgNPs synthesized from the three distinct green tea extracts, which correlates with their identical MIC values (Table 1 and Figure 7). Concerning ROS production induced by AgNPs generated either from coffee or Virginia creeper extracts, it was observed that the AgNPs derived from the first extracts produced the lowest levels of ROS, with a visible increase in ROS levels toward the third extracts; however, this variation was not statistically significant (Figure 7). Nonetheless, this alteration in ROS levels may account for the differences in their respective MIC values.

**Figure 7:**
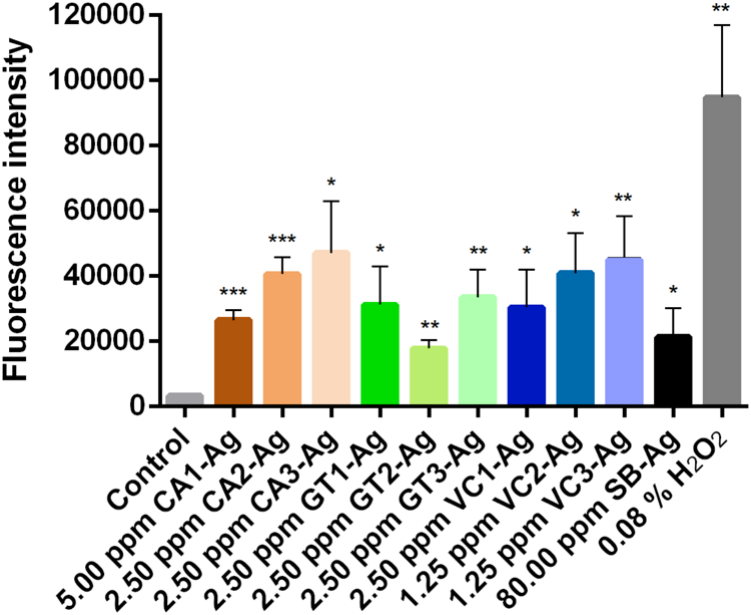
Formation of reactive oxygen species in *C. neoformans* after AgNP treatment at half-MIC concentration detected by DCF fluorescence. The intensity of DCF fluorescence was quantified in DCFDA-stained *C. neoformans* cells that were treated with AgNPs. Untreated, DCFDA-stained cells served as the negative control, while H_2_O_2_-treated DCFDA-stained cells functioned as the positive control. Values represent the mean ± standard deviation calculated from three independent experiments (***: *P* < 0.001, **: *P* < 0.005 *: *P* < 0.05; unpaired *t* test). CA1-Ag, CA2-Ag, CA3-Ag: AgNPs synthesized by 1st, 2nd or 3rd coffee extract; VC1-Ag, VC2-Ag, and VC3-Ag: AgNPs synthesized by 1st, 2nd or 3rd round Virginia creeper extract; GT1-Ag, GT2-Ag, and GT3-Ag: AgNPs synthesized by 1st, 2nd or 3rd green tea extract. SB-Ag: AgNP synthesized by chemical method using sodium borohydride.

### 3.7 Release of Silver Ions

To explore the possible explanations behind the differences in biological activity between AgNPs that were prepared with CA, VC, and GT extracts from subsequent extraction rounds, the amount of silver ions released from each type of nanoparticle was determined using ICP-MS analysis. According to the results, the concentration of dissolved silver was 0.91 mM for SB-Ag, 0.63, 0.95, and 1.12 mM for CA1-Ag, CA2-Ag, and CA3-Ag, respectively; 0.65, 0.69, and 0.87 mM for GT1-Ag, GT2-Ag, and GT3-Ag, respectively; and 0.72, 0.85, and 0.96 mM for VC1-Ag, VC2-Ag, and VC3-Ag, respectively (Figure 8). In all cases, an increasing trend was observed in the silver ion release across the three extraction rounds.

**Figure 8:**
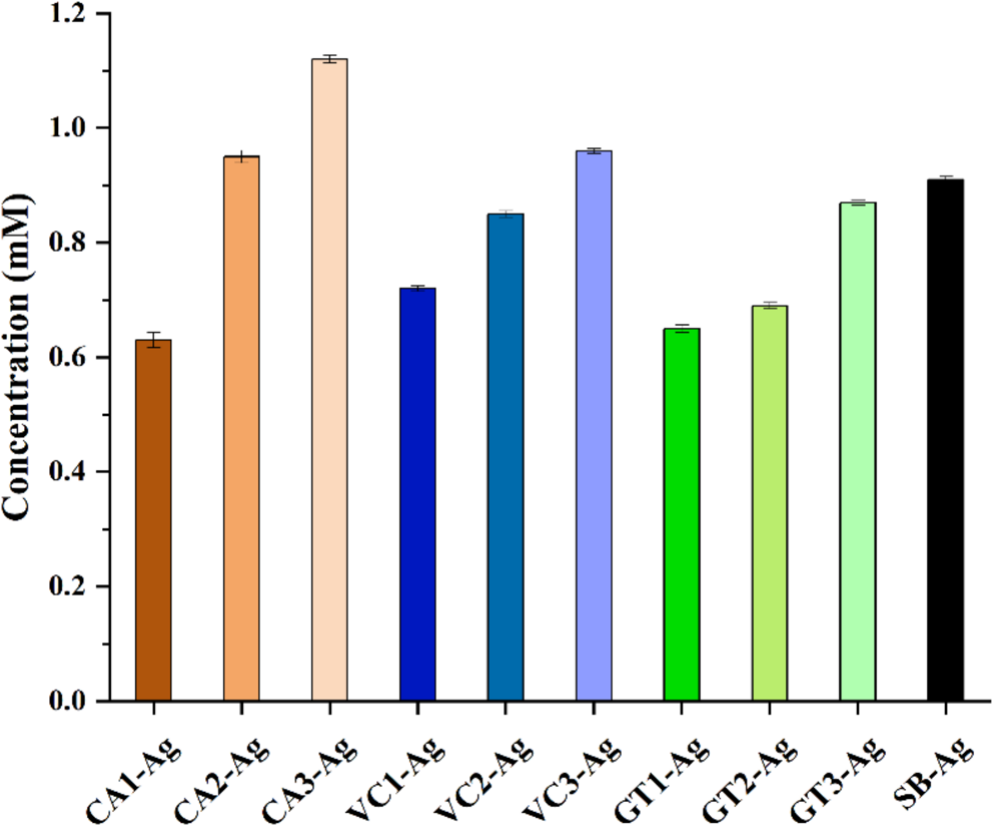
Ag^+^ ion concentration obtained by ICP-MS of AgNPs produced with various green waste extracts of three extraction rounds. Abbreviations stand for AgNPs generated by 1st, 2nd, and 3rd extracts of coffee: CA1-Ag, CA2-Ag, CA3-Ag, Virginia creeper: VC1-Ag, VC2-Ag, and VC3-Ag, green tea: GT1-Ag, GT2-Ag, and GT3-Ag, and in a chemical method using sodium borohydride: SB-Ag.

## 4 Discussion

The application of nanoparticles in modern medicine has been substantially expanded to a wide range of healthcare approaches, particularly in clinical microbiology and cancer therapy. Especially metallic nanoparticles such as AgNPs represent promising alternatives to traditional pharmaceuticals as antimicrobial and anticancer agents; their potential is currently being explored in greater detail.^24,25^ The expanding demand for AgNPs calls for the development of environmentally friendly, cost-effective methods, and innovations in synthesis techniques, with a focus on green chemistry principles to minimize waste and reduce energy consumption, to yield biogenic AgNPs in large quantities.^26^

Accordingly, in our study, we aimed to examine the suitability and reusability of various green household plant waste extracts, subjected to multiple extraction rounds, for the synthesis of silver nanoparticles. We intended to investigate the potential of the produced AgNPs in medicine; therefore, their physicochemical properties and their cytotoxicity against Gram-positive and Gram-negative bacteria, fungi, and also human cancerous and noncancerous cells were evaluated. For this, three different plant-derived materials, namely powdered coffee arabica, Virginia creeper leaves, and dry green tea leaves were collected and extracted (CA1, VC1, and GT1). The waste materials that were left behind at the end of the extractions were dried and recycled in two additional extraction rounds to yield individual extracts each time, labeled as CA2, CA3 for coffee, VC2, and VC3 of Virginia creeper, and GT2, and GT3 for green tea, respectively. Then, each extract was subjected to AgNP synthesis. Comprehensive chemical and biological characterization of the obtained nanoparticles demonstrated that the green synthesis using extracts from subsequent extraction rounds results in distinct variations in particle size, stability, and biological activity. The workflow depicted in Figure 9A demonstrates the steps starting from the multi-round green synthesis that leverages plant waste extracts in a resource-efficient manner, leading to biologically effective nanoparticle production. The present results were also consistent with our previous findings, where we showed that the various green materials used for the stabilization and reduction of metal ions play a crucial role in determining and fine-tuning the biological activity of the resulting nanoparticles.^17^

**Figure 9:**
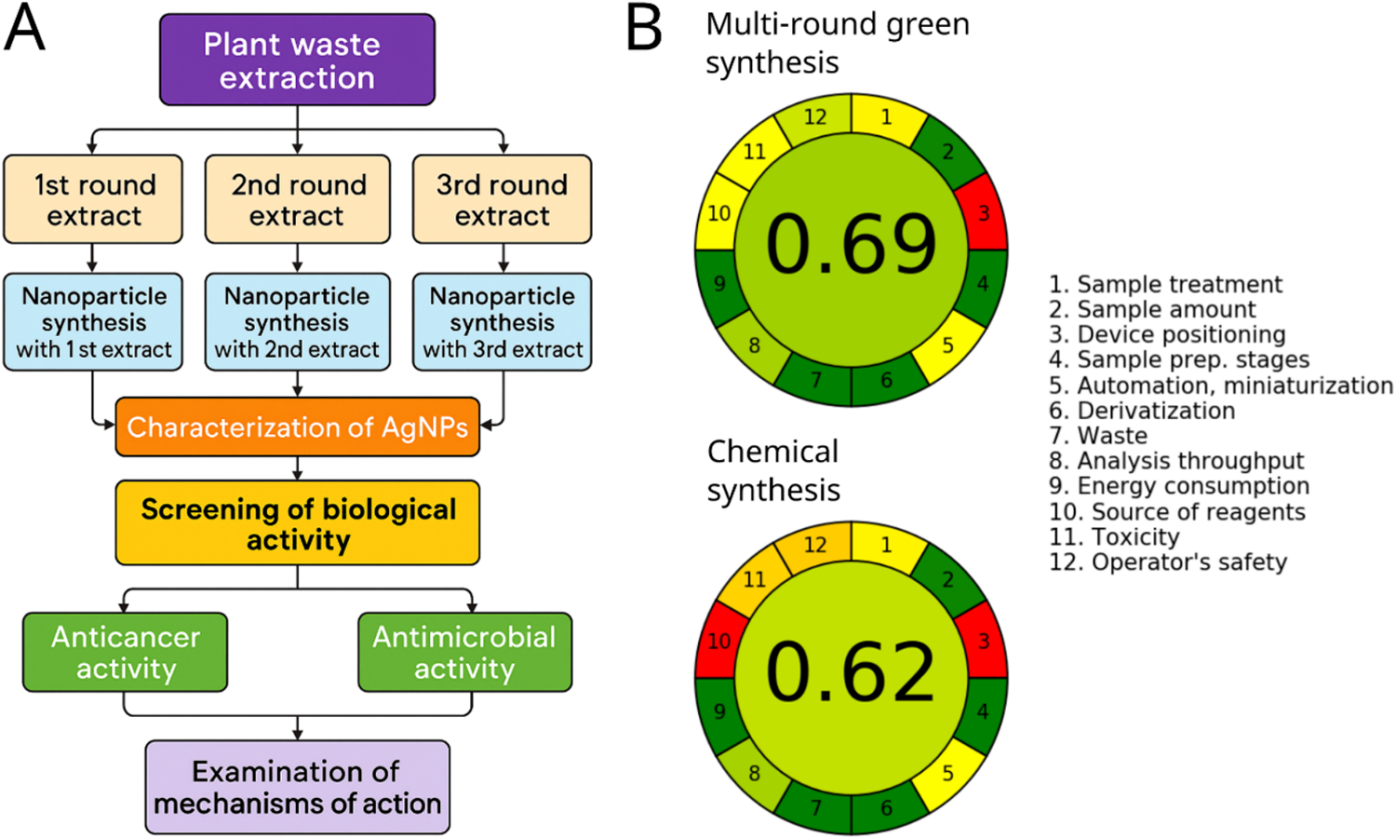
Workflow of multi-round plant waste extraction, nanoparticle synthesis, and bioactivity evaluation (A) and AGREE (Analytical GREEnness Metric Approach and Software) results for assessing the “greenness” of the developed multi-round method (B).

Although fresh plant materials were used in this work to prepare the first extracts, the extraction and synthesis protocol is readily adaptable to postconsumer or food-processing plant waste, such as spent tea leaves, used coffee grounds, or dried herb residues. With minimal preprocessing (such as washing and drying), these materials retain sufficient phytochemical content to reduce and stabilize silver ions effectively. This highlights the broader potential of the method in real-world circular bioeconomy applications. As a brief qualitative sustainability assessment, we evaluated our approach based on several green chemistry principles,^27^ including reduced energy and chemical usage, minimized waste generation, and improved biomass utilization efficiency. This approach was specifically designed to maximize the extraction and utilization of phytochemicals from a single biomass input, enhancing sustainability. Indeed, the novelty of this work is the multi-round green synthesis of silver nanoparticles (AgNPs) using repeated extractions from the same batch of plant leaves instead of using new material each time. For energy savings, traditional one-time extraction requires fresh plant material to be collected and processed (including washing, drying, homogenizing, and heating) for each synthesis cycle, as the bioactive compounds responsible for nanoparticle reduction and stabilization are typically utilized only once, which consumes significant energy. In contrast, in our method, the same plant material was used across three successive extraction rounds, avoiding repeated biomass preparation steps, thus significantly reducing energy input per synthesis batch. In terms of chemical usage, while green synthesis is already beneficial in avoiding toxic reagents, our method further minimizes chemical input and supports a more sustainable footprint by maximizing the use of eco-friendly reducing agents across all rounds. No additional chemicals were used beyond the silver nitrate precursor and water as solvent. Notably, since the same biomass was extracted multiple times, there was no need for extra reducing agents or stabilizers in each round, in contrast to one-time methods where fresh biomass is used per batch, thereby increasing chemical consumption. For waste reduction metrics, in typical green synthesis protocols, the plant material is discarded after a single extraction, despite still containing unextracted bioactive compounds. By performing three successive extractions from the same leaves, our approach significantly enhanced biomass usage and reduced waste generation (app. 66% as one plant sample yields three batches instead of one). In summary, our multi-round biosynthesis improves the efficiency of biomass utilization, minimizes chemical and energy inputs, and significantly reduces waste. It aligns with green chemistry principles and offers a sustainable, circular bioeconomy model for nanoparticle synthesis. Furthermore, while our focus was on feasibility and bioactivity, we also estimated nanoparticle production yields. Each gram of reused plant biomass contributed to approximately 10–15 mg of AgNP formation during later extraction rounds, suggesting a relatively efficient conversion of phytochemical reducing agents into functional nanomaterials. Triplicate syntheses were conducted for all samples, and results across nanoparticle size, ζ-potential, UV–vis spectra, and biological activity showed low variability, supporting good reproducibility at the laboratory scale. Further study will include precise quantification of nanoparticle recovery efficiency and expanded batch-to-batch reproducibility using independently sourced biomass. Future work will also focus on incorporating these green-synthesized AgNPs into air filtration media and evaluating their retention, stability under airflow, and silver ion release dynamics under realistic environmental conditions. Although our current protocol operates at moderate temperatures (70 °C), further optimization toward low-energy synthesis is essential for industrial scalability. The use of bioderived ligands in plant extracts may already contribute to energy efficiency by enabling nanoparticle formation under relatively mild conditions, aligning with recent advances in low-energy catalytic systems.^28^ The Analytical GREEnness Metric Approach and Software helped to assess the ″greenness″ of our multi-round waste utilizing AgNP method by providing a quantitative metric to evaluate the environmental friendliness of the technique and facilitate comparison with other synthetic approaches.^29^ The AGREE evaluation in Figure 9B demonstrates that the multi-round green synthesis achieved a higher greenness score (0.69) compared to the conventional chemical synthesis method (0.62), emphasizing further reduced toxicity, renewable reagent sourcing, and improved waste management of our green method.

Following nanoparticle syntheses, TEM analysis was performed, confirming that all multi-round recycled green waste-mediated AgNPs formed with isotropic morphology. Interestingly, the particle sizes did not change in such a way as it was expected, and different trends were observed for all three plant-derived materials. While the size of AgNPs synthesized by using VC extracts increased with the number of extraction rounds, size reductions were observed in nanoparticles synthesized with CA extracts of the second and third extraction rounds. In contrast, when GT extracts were used, the particle sizes remained nearly similar across the syntheses using extracts from all three rounds. This correlated only partially with the observations made during the synthesis of other metal nanoparticles using the same multi-recycled plant extracts.^18^ In those cases, the particle size increased with each extraction round for each plant.

This increase was attributed to the decreasing concentration of bioactive agents, such as polyphenols, tannins, catechins, amino acids, enzymes, vitamins, and reducing sugars, which facilitate particle generation in metal ion-supplemented media, since these biodegradable, nontoxic substances serve as both reducing and capping agents. Especially phenolic compounds and reducing sugars from natural sources, due to their ability to protonate and absorb, along with their antioxidant properties, have crucial roles, because their strong oxidation potential can initiate nucleation, reduce metal salts to nanoparticles, enhance colloid stability, and prevent agglomeration through physical attachment to particle surfaces, as confirmed by infrared spectroscopic analysis.^12,17,30,31^ It was also demonstrated that the above-mentioned reducing substances were extracted from the green waste in several rounds; therefore, their quantity was probably sufficient in each round extract to yield NPs. Nevertheless, the reduced presence of these biomolecules in subsequent extracts resulted in larger nanoparticles due to less efficient reduction and capping, leading to particle growth.^18^ In the present work, particle size decreased in CA extracts across rounds, possibly due to early round stabilizer loss, while VC extracts showed increased size in the second round, consistent with reduced capping efficiency. GT extracts maintained small, stable particles, suggesting consistent phytochemical strength. These variations confirm that the extract composition evolves with repeated use, directly affecting nanoparticle characteristics. The XRD patterns confirmed that crystalline AgNPs were formed in all extraction rounds, but the decreasing peak intensities with successive extracts suggest reduced phytochemical availability affecting nanoparticle crystallinity. The emergence of minor peaks in later rounds (especially in CA3-Ag) may indicate the presence of residual organics or partial silver oxidation, reflecting changes in extract composition and capping efficiency.^16^ It should be noted that XRD measurements were performed using Co Kα radiation (λ = 1.78897 Å), which leads to a systematic shift in 2θ values. In parallel, the observed reduction both in AgNP yield and UV–vis absorbance intensity with each extraction round indicates a decline in extract potency. Although the phytochemical content was not quantified in this study, our previous findings demonstrated a significant drop in total phenolic and sugar contents after each successive extraction.^18^ These reductions in chemical yield likely explain the weakened reducing and stabilizing capacity of the reused extracts, ultimately affecting both nanoparticle formation efficiency and properties. This interpretation aligns with recent findings that phenolic compounds not only cap and stabilize AgNPs but also modulate their antimicrobial efficacy.^32^

The balance between nucleation and stabilization during synthesis influenced significantly the size of nanoparticles; this intricate and rather sensitive equilibrium determines the nanoparticle dimensions.^33,34^ Generally, surfactants can prevent particle growth by capping the surface of nanoparticles, and their dilution allows better control of the particle size.

In green synthesis approaches, where plant extracts or microorganisms are applied, these factors become even more critical, given that these materials contain numerous natural surfactants as well. Therefore, both the concentration of reducing agents and the surfactant-driven stabilization process must be carefully managed, since these dual mechanisms of reduction-capping and nucleation**-**stabilization have significant, mainly opposite effects on nanoparticle morphology and stability.^35,36^ According to DLS measurements, a decrease in hydrodynamic size and ζ-potential value was observed for all samples, indicating a gradual loss of colloidal stability, likely due to diminishing levels of natural capping agents. This observation aligns with earlier findings that nanoparticles with smaller sizes, narrower hydrodynamic size distributions, and less negative ζ-potentials tend to be less stable and more prone to aggregation and accumulation.^20^ To mitigate these challenges, postsynthetic surface modifications using biocompatible coatings such as chitosan, alginate, or polyethylene glycol (PEG) or by embedding AgNPs into support matrices (e.g., cellulose, fibers) may ensure long-term stability under operational conditions.^37^

The somewhat decreased nanoparticle stability is also reflected in our UV–vis spectroscopic analysis, where both the intensity and position of the surface plasmon resonance (SPR) peak vary across rounds (Figure 4). This can be attributed primarily to a lower concentration of reducing and capping agents in the reused plant extracts. Notably, a blue shift in the SPR peak of all green samples suggests the formation of smaller or more aggregated particles, while the drop in peak intensity indicates a lower overall yield of well-formed AgNPs. This likely results from differential retention of capping agents, consistent with our earlier FTIR compositional analysis showing depletion of bioactive phytochemicals. In contrast, chemically synthesized AgNPs showed a sharp SPR peak, characteristic of smaller, more uniform nanoparticles. This comparison highlights the distinct role of plant-derived phytochemicals in modulating nanoparticle size, uniformity, and yield. Our ICP-MS measurements further support this interpretation as silver ion release increased with each extraction round (e.g., CA1-Ag: 0.63 mM → CA3-Ag: 1.12 mM), implying that nanoparticles from later extracts have lower stability. Since all samples were thoroughly washed, the detected Ag^+^ likely originated from weakly bound surface silver, not residual AgNO_3_. Furthermore, reduced stabilization can promote aggregation, leading to enhanced light scattering, which distorts the UV–vis spectrum and further diminishes the apparent absorbance.^38^ These results suggest that extract composition evolution directly influences nucleation and growth kinetics, modulating both particle size and stability; nevertheless, given the plethora of organic molecules found in each extract, determining which of these defines the utmost nanoparticle dimensions requires a comprehensive multimodal technical approach, which is beyond the limits and goals of the present work.

These chemical properties naturally affect the biological behavior and potential biological applications of the nanoparticles. Therefore, the other aim of our study was to investigate the performance and biomedical suitability of green AgNPs produced with recycled plant waste materials. We found that all of the AgNPs, particularly those generated by extracts from the second and third extraction rounds, were highly toxic to a range of rather problematic cancer types. All of these AgNPs caused significant cell death in A549 radiation-resistant lung, MCF-7 breast, MCF-7 KCR multidrug-resistant breast, as well as MDA-MB-231 triple-negative breast adenocarcinoma cells, proving that the nanoparticles generated by green plant waste extracts exhibit potent anticancer activity. However, cancer-selective toxicity could not be verified, as the green AgNPs manifested similar cytotoxicity levels in both cancerous and noncancerous cells. According to our results, AgNPs do not induce necrosis in human cells, indicating another form of cell death behind the observed viability decrease. Many studies revealed that green-synthesized AgNPs induce ROS-mediated apoptosis in cancer cells by the activation of caspase enzymes,^39,40^ p53 protein,^41^ and upregulation of apoptotic marker genes.^42^

Regarding the antimicrobial activity, the synthesized AgNPs proved to be highly effective against various microbial strains, with GT-AgNPs exhibiting the strongest antimicrobial activity against *B. megaterium*, while CA-AgNPs were most effective against *E. coli* and VC-AgNPs, achieved the best results against *A. niger* and *C. neoformans*. Interestingly, a trend of higher antibacterial and antifungal efficacy was identified for AgNPs that were generated with extracts of later extraction rounds. These results correlate with our and others’ previous studies, proving the efficient antibacterial and antifungal properties of green-synthesized AgNPs against pathogens and multidrug-resistant species using different plant extracts,^17,43,44^ or microorganisms.^45,46^ In our previous study, we showed that green-synthesized AgNPs (using *Phaffia rhodozyma* for the synthesis) significantly inhibited the growth of opportunistic pathogen *Candida*, and *Cryptococcus* species, and were highly efficient against *Microsporum* and *Trichophyton* dermatophytes^45^ but did not affect the viability of human keratinocyte cells. Moreover, we have proven that the natural extract applied upon green synthesis remarkably influences the biological activity of AgNPs since coffee extract-based and green tea extract-based AgNPs exhibited high antimicrobial activity against bacterial and yeast pathogens, but only green tea AgNPs induced anticancer activity and showed high toxicity to noncancerous mammalian cells.^17^ The observed differences in the cytotoxicity of AgNPs can be explained by several physical aspects, such as the size, charge, or morphology of nanoparticles, and the matrix coming from the applied green extract. Since smaller nanoparticles possess higher toxicity due to their larger relative surface area, which enables the more intensive release of reactive silver ions.^17^ Several other studies have verified that the dimensions and morphology of AgNPs are significant determinants of their biological activity.^47,48^ Cheon et al. reported that the antimicrobial efficacy of silver nanoparticles exhibited a dependence on their shape, whereas the morphology did not influence cytotoxicity, considering also the distinct interactions between AgNPs and mammalian as well as bacterial cells.^49^ Furthermore, the formation of a multicomponent matrix on the surface of AgNPs can also influence their silver ion-releasing capabilities, thereby ultimately affecting the biological activity. Indeed, it was shown that behind the antimicrobial activity, there is microbial membrane damage, induced by the attachment of AgNPs on the cell surface, which results in structural and functional alterations in the membrane, such as destabilization, formation of pores, and cytoplasm leakage. In addition, the release of Ag ions from the nanoparticle surface provokes the generation of reactive oxygen species, leading to subcellular structure damage and inactivation of essential microbial macromolecules, causing ultimately apoptotic cell death.^50^ In fact, we proved that all of the green AgNPs induced significant ROS formation in the tested microbial samples. The cytotoxicity against mammalian cells is triggered by similar events; the primary molecular mechanisms comprise silver ion-mediated destruction of the cell membrane, the inhibition of ATP synthesis, reactive oxygen species (ROS) generation and apoptosis or other cell death induction.^29^ Based on our finding that none of these green AgNPs caused necrosis in the tested mammalian cell line, it is plausible that their strong cytotoxic effect, seen as a loss of viability, is the result of apoptotic cell death triggered by reactive silver ions, ROS and oxidative stress.

The release of silver ions from nanoparticles is a critical factor influencing their biological activity. We observed differences in the silver ion-releasing capability of the AgNPs that were consistent with the variations in nanoparticle stability and size. VC-mediated AgNPs demonstrated the most significant antifungal activity, possibly due to the higher silver ion release, which is known to enhance toxicity in microbial cells. The increase in silver ion release and ROS generation with each extraction round in VC samples suggests a correlation between the reduced nanoparticle stability and the high biological activity of the VC-AgNPs observed in antimicrobial assays. For instance, the highest silver ion release of the third round extract-mediated AgNPs could be due to a gradual depletion of stabilizing agents like polyphenols and catechins, which act as capping agents in earlier rounds.^18^ As the nanoparticles become less effectively capped, an increasing trend in silver ion release can be observed. Such a trend is somewhat less pronounced for CA- and GT-mediated AgNPs, where the increase in silver ion release is more moderate. This may be due to differences in the bioactive compound profiles of coffee arabica and green tea extracts. Further fine-tuning of the extraction and synthesis process could help achieve an optimal balance between ion release and particle stability.

The utilization of such multi-round green waste-mediated AgNPs in cancer treatment faces challenges, particularly due to the lack of cancer cell selectivity; nevertheless, the antimicrobial efficacy of these AgNPs can be fully exploited in alternative applications. One promising application is in air purification systems, where the demand for efficient, cheap, and sustainable antimicrobial solutions is ever-growing. Air conditioning systems, especially in densely populated environments, can act as reservoirs and dissemination points for airborne pathogens, posing significant public health risks.^51^ While High-Efficiency Particulate Air (HEPA) filters are effective at trapping microorganisms, they often lack inherent antimicrobial properties, increasing the risk of microbial proliferation and contamination. By incorporating antimicrobial agents such as AgNPs into air filters, the risks associated with trapped pathogens can be significantly mitigated. Our study showed that green-synthesized AgNPs, produced using multi-round extractions of all three types of waste, exhibited strong antimicrobial activity against a broad spectrum of bacterial and fungal pathogens. Antimicrobial efficacy increased with subsequent extraction rounds, a trend that correlated with increased silver ion release. The sustained release of silver ions observed in these AgNPs is particularly advantageous for air filtration applications, where prolonged antimicrobial effects are necessary to prevent microbial growth on filter surfaces. The incorporation of green-synthesized AgNPs into air conditioning filters offers a dual advantage: improving air quality by neutralizing airborne pathogens and reducing environmental organic waste through sustainable production methods. Beyond biomedical and antimicrobial applications, the recovered silver nanoparticles may also serve as catalyst promoters in energy-related processes,^52^ highlighting their potential in sustainable chemical technologies. Moreover, the multi-round green synthesis concept could be adapted for the recovery and reuse of other noble metals such as gold or palladium, expanding the scope of this method for industrial catalysis, electronic materials, or environmental remediation. Further work will be needed to optimize these recovery protocols for scalability and performance in catalytic systems.

Our results demonstrated that CA, VC, and GT green wastes are suitable for multi-round extractions, and the repeated reuse of plant extracts enhances the sustainability and cost-effectiveness of AgNPs synthesis, making it a viable alternative to conventional synthesis methods. However, further research is needed to optimize the adhesion of AgNPs to filter materials, evaluate their performance under real-world conditions, and ensure long-term stability and safety. Exploring additional plant waste materials and refining the synthesis process could further enhance the efficacy and scalability of this approach. The project and the results are also aligned with key United Nations Sustainable Development Goals by advancing a green, waste-based method for silver nanoparticle synthesis that supports sustainable production, improved public health, and climate-responsible innovation through scalable antimicrobial applications.

## Conclusions

This study demonstrated the successful and reproducible synthesis of silver nanoparticles (AgNPs) using *C. arabica*, green tea, and Virginia creeper waste extracts subjected to up to three sequential extraction cycles. The physicochemical and biological profiles of the resulting AgNPs varied notably across recycling rounds, with later extracts yielding nanoparticles of smaller size, reduced colloidal stability, and elevated silver ion release—properties that translated into enhanced antimicrobial and cytotoxic potency. All green-synthesized AgNPs exhibited robust broad-spectrum antimicrobial activity and significant cytotoxic effects against various human cancer cell lines, although lacking tumor specificity. Importantly, the progressive increase in reactive oxygen species (ROS) generation and silver ion release in later-round AgNPs correlated with diminishing phytochemical capping and stabilization, confirming a mechanistic link between nanoparticle composition, redox activity, and bioefficacy. These findings substantiate that the repeated reuse of plant waste extracts leads to evolving extract compositions that govern nanoparticle characteristics—such as size, ζ-potential, ion release capacity, and ROS-inducing potential—which collectively determine biological outcomes. Notably, nanoparticles synthesized in later rounds (CA3-, GT3-, VC3-AgNPs) consistently outperformed those from initial extracts in both antibacterial (MIC as low as 0.62 ppm) and antifungal assays, and displayed potent cytotoxicity across lung and breast cancer cell lines, including multidrug-resistant and triple-negative subtypes.

The environmentally benign, scalable, and waste-minimizing nature of this green synthesis protocol, combined with the demonstrated biological efficacy of the resulting nanomaterials, underscores their significant industrial potential—particularly in nontherapeutic applications such as antimicrobial coatings and air purification systems. This approach offers a dual advantage: effective valorization of agricultural and household plant waste, and the production of biologically active nanomaterials aligned with green chemistry principles. Future efforts will aim to optimize nanoparticle immobilization on functional surfaces, evaluate performance under operational conditions, and further explore the circular utility of diverse waste biomass for sustainable nanotechnology.

## Supplementary Material



## Notes

The viability of (A) A549, (B) MCF-7, (C) MCF-7 KCR, (D) MDA-MB-231 cancerous, and (E) MRC-5 noncancerous cells 24 h after AgNP-free green waste extract (CA, VC, GT) treatments (Figure S1); growth of *E. coli*, *B. megaterium*, *A. niger*, and *C. neoformans* after AgNP-treatment (Figure S2); minimal inhibitory concentration of multi-round generated green waste extracts against bacteria (*E. coli* and *B. megaterium*) and fungi (*A. niger* and *C. neoformans*) (Table S1) (PDF)

## References

[1] Chavali M. S., Nikolova M. P. (2019). Metal oxide nanoparticles and their applications in
nanotechnology. SN Appl. Sci..

[2] Shnoudeh A. J., Hamad I., Abdo R. W., Qadumii L., Jaber A. Y., Surchi H. S., Alkelany S. Z. (2019). Synthesis,
Characterization, and
Applications of Metal Nanoparticles. Biomater.
Bionanotechnol..

[3] Bourang S., Noruzpour M., Godekahriz S. J., Ebrahimi H. A. C., Amani A., Zakaria R. A., Yaghoubi H. (2024). Application of nanoparticles in breast
cancer treatment: a systematic review. Naunyn-Schmiedeberg’s
Arch. Pharmacol..

[4] Kuchur O. A., Tsymbal S. A., Shestovskaya M. V., Serov N. S., Dukhinova M. S., Shtil A. A. (2020). Metal-derived nanoparticles
in tumor theranostics:
Potential and limitations. J. Inorg. Biochem..

[5] Naganthran A., Verasoundarapandian G., Khalid F. E., Masarudin M. J., Zulkharnain A., Nawawi N. M. (2022). Synthesis, Characterization
and Biomedical Application of Silver Nanoparticles. Materials.

[6] Zhang X. F., Liu Z. G., Shen W., Gurunathan S. (2016). Silver nanoparticles:
Synthesis, characterization, properties, applications, and therapeutic
approaches. Int. J. Mol. Sci..

[7] Miranda R. R., Sampaio I., Zucolotto V. (2022). Exploring
silver nanoparticles for
cancer therapy and diagnosis. Colloids Surf.,
B.

[8] Yang R., Chen L., Wang Y., Zhang L., Zheng X., Yang Y., Zhu Y. (2023). Tumor microenvironment
responsive
metal nanoparticles in cancer immunotherapy. Front. Immunol..

[9] Abdolahinia E. D., Fathi M., Pirdel Z., Jafari S., Samiei M., Adibkia K. (2022). Strategies to improve
drug penetration into tumor microenvironment
by nanoparticles: Focus on nanozymes. OpenNano.

[10] Sell M., Lopes A. R., Escudeiro M., Esteves B., Monteiro A. R., Trindade T., Cruz-Lopes L. (2023). Application
of Nanoparticles in Cancer
Treatment: A Concise Review. Nanomaterials.

[11] Dawadi S., Katuwal S., Gupta A., Lamichhane U., Thapa R., Jaisi S. (2021). Current
Research on
Silver Nanoparticles: Synthesis, Characterization, and Applications. J. Nanomater..

[12] Rónavári A., Igaz N., Adamecz D. I., Szerencsés B., Molnar C., Kónya Z. (2021). Green silver and gold
nanoparticles: Biological synthesis approaches and potentials for
biomedical applications. Molecules.

[13] Moghaddam A. B., Namvar F., Moniri M., Tahir P. M., Azizi S., Mohamad R. (2015). Nanoparticles biosynthesized by fungi
and yeast: A
review of their preparation, properties, and medical applications. Molecules.

[14] Ebrahiminezhad A., Zare-Hoseinabadi A., Sarmah A. K., Taghizadeh S., Ghasemi Y., Berenjian A. (2018). Plant-Mediated
Synthesis and Applications
of Iron Nanoparticles. Mol. Biotechnol..

[15] Rodrigues M. C., Rolim W. R., Viana M. M., Souza T. R., Gonçalves F., Tanaka C. J. (2020). Biogenic
synthesis and antimicrobial activity
of silica-coated silver nanoparticles for esthetic dental applications. J. Dent..

[16] Mittal A. K., Chisti Y., Banerjee U. C. (2013). Synthesis of metallic
nanoparticles
using plant extracts. Biotechnol. Adv..

[17] Rónavári A., Kovács D., Igaz N., Vágvölgyi C., Boros I. M., Kónya Z. (2017). Biological activity
of green-synthesized silver nanoparticles depends on the applied natural
extracts: A comprehensive study. Int. J. Nanomed..

[18] Rónavári A., Balázs M., Szilágyi Á., Molnár C., Kotormán M., Ilisz I. (2023). Multi-round recycling
of green waste for the production of iron nanoparticles: synthesis,
characterization, and prospects in remediation. Discover Nano.

[19] Kozma G., Rónavári A., Kónya Z., Kukovecz Á. (2016). Environmentally Benign Synthesis
Methods of Zero-Valent
Iron Nanoparticles. ACS Sustainable Chem. Eng..

[20] Bélteky P., Rónavári A., Igaz N., Szerencsés B., Tóth I. Y., Pfeiffer I. (2019). Silver nanoparticles:
Aggregation behavior in biorelevant conditions and its impact on biological
activity. Int. J. Nanomed..

[21] Patil M. P., Kim G.-D. (2017). Eco-friendly approach
for nanoparticles synthesis and
mechanism behind antibacterial activity of silver and anticancer activity
of gold nanoparticles. Appl. Microbiol. Biotechnol..

[22] Bélteky P., Rónavári A., Zakupszky D., Boka E., Igaz N., Szerencsés B. (2021). Are Smaller Nanoparticles Always Better? Understanding the Biological
Effect of Size-Dependent Silver Nanoparticle Aggregation Under Biorelevant
Conditions. Int. J. Nanomed..

[23] Rónavári A., Bélteky P., Boka E., Zakupszky D., Igaz N., Szerencsés B. (2021). Polyvinyl-pyrrolidone-coated
silver nanoparticles—The colloidal, chemical and biological
consequences of steric stabilization under biorelevant conditions. Int. J. Mol. Sci..

[24] Chandrakala V., Aruna V., Angajala G. (2022). Review on metal nanoparticles as
nanocarriers: current challenges and perspectives in drug delivery
systems. Emergent Mater..

[25] Rai M., Kon K., Ingle A., Duran N., Galdiero S., Galdiero M. (2014). Broad-spectrum
bioactivities of silver nanoparticles: The emerging trends and future
prospects. Appl. Microbiol. Biotechnol..

[26] Hembram K. C., Kumar R., Kandha L., Parhi P. K., Kundu C. N., Bindhani B. K. (2018). Therapeutic prospective
of plant-induced silver nanoparticles:
application as antimicrobial and anticancer agent. Artif. Cells, Nanomed., Biotechnol..

[27] Osman A. I., Zhang Y., Farghali M., Rashwan A. K., Eltaweil A. S., El-Monaem E. M. A. (2024). Synthesis of green nanoparticles
for energy,
biomedical, environmental, agricultural, and food applications: a
review. Environ. Chem. Lett..

[28] Osman A. I., Ayati A., Krivoshapkin P., Tanhaei B., Farghali M., Yap P. S., Abdelhaleem A. (2024). Coordination-driven
innovations in
low-energy catalytic processes: Advancing sustainability in chemical
production. Coord. Chem. Rev..

[29] Pena-Pereira F., Wojnowski W., Tobiszewski M. (2020). AGREE—Analytical
GREEnness
metric approach and software. Anal. Chem..

[30] Dhand V., Soumya L., Bharadwaj S., Chakra S., Bhatt D., Sreedhar B. (2016). Green synthesis of silver nanoparticles using *Coffea arabica* seed extract and its antibacterial
activity. Mater. Sci. Eng., C.

[31] Loo Y. Y., Chieng B. W., Nishibuchi M., Radu S. (2012). Synthesis of silver
nanoparticles by using tea leaf extract from *Camellia
Sinensis*. Int. J. Nanomed..

[32] Scroccarello A., Molina-Hernández B., Pelle F. D., Ciancetta J., Ferraro G., Fratini E. (2021). Effect of phenolic compounds-capped
AgNPs on growth inhibition of *Aspergillus niger*. Colloids Surf., B.

[33] Xu L., Liang H. W., Yang Y., Yu S. H. (2018). Stability and Reactivity:
Positive and Negative Aspects for Nanoparticle Processing. Chem. Rev..

[34] Thanh N. T. K., Maclean N., Mahiddine S. (2014). Mechanisms
of nucleation and growth
of nanoparticles in solution. Chem. Rev..

[35] Khan M. N., Khan T. A., Khan Z., AL-Thabaiti S. A. (2015). Green synthesis
of biogenic silver nanomaterials using *Raphanus sativus* extract, effects of stabilizers on the morphology, and their antimicrobial
activities. Bioprocess Biosyst. Eng..

[36] Lvov, Y. Handbook of Surfaces and Interfaces of Materials. In Nanostructured Materials, Micelles and Colloids; Academic Press, 2001; Vol. 3, pp 170–189.

[37] Satchanska G., Davidova S., Petrov P. D. (2024). Natural and synthetic polymers for
biomedical and environmental applications. Polymers.

[38] Achilefu, S. ; Raghavachari, R. In Reporters, Markers, Dyes, Nanoparticles, and Molecular Probes for Biomedical Applications X, Proceedings of SPIE; SPIE: San Francisco, CA, USA. Bellingham, WA, 2018; pp 1–112.

[39] Ullah I., Khalil A. T., Ali M., Iqbal J., Ali W., Alarifi S., Shinwari Z. K. (2020). Green-Synthesized
Silver Nanoparticles
Induced Apoptotic Cell Death in MCF-7 Breast Cancer Cells by Generating
Reactive Oxygen Species and Activating Caspase 3 and 9 Enzyme Activities. Oxid. Med. Cell. Longevity.

[40] Lalsangpuii F., Rokhum S. L., Nghakliana F., Ruatpuia J. V. L., Tochhawng L., Trivedi A. K., Lalfakzuala R., Siama Z. (2024). *Mikania
micrantha* silver nanoparticles exhibit anticancer
activities against human lung adenocarcinoma via caspase-mediated
apoptotic cell death. Artif. Cells, Nanomed.,
Biotechnol..

[41] Simsek A., Pehlivanoglu S., Acar C. A. (2021). Anti-proliferative and apoptotic
effects of green synthesized silver nanoparticles using *Lavandula angustifolia* on human glioblastoma cells. 3 Biotech.

[42] Al-Asiri W. Y., Al-Sheddi E. S., Farshori N. N., Al-Oqail M. M., Al-Massarani S. M., Malik T. (2024). Cytotoxic and Apoptotic Effects of Green Synthesized
Silver Nanoparticles via Reactive Oxygen Species–Mediated Mitochondrial
Pathway in Human Breast Cancer Cells. Cell Biochem.
Funct..

[43] Ibrahim E. H., Kilany M., Ghramh H. A., Khan K. A., ul Islam S. (2019). Cellular proliferation/cytotoxicity
and antimicrobial potentials of green synthesized silver nanoparticles
(AgNPs) using *Juniperus procera*. Saudi J. Biol. Sci..

[44] Senthil B., Devasena T., Prakash B., Rajasekar A. (2017). Non-cytotoxic
effect of green synthesized silver nanoparticles and its antibacterial
activity. J. Photochem. Photobiol., B.

[45] Rónavári A., Igaz N., Gopisetty M. K., Szerencsés B., Kovács D., Papp C. (2018). Biosynthesized silver
and gold nanoparticles are potent antimycotics against opportunistic
pathogenic yeasts and dermatophytes. Int. J.
Nanomed..

[46] Abishad P., Vergis J., Unni V., Ram V. P., Niveditha P., Yasur J. (2022). Green Synthesized Silver Nanoparticles Using *Lactobacillus Acidophilus* as an Antioxidant, Antimicrobial,
and Antibiofilm Agent Against Multi-drug Resistant Enteroaggregative *Escherichia Coli*. Probiotics
Antimicrob. Proteins.

[47] Pal S., Tak Y. K., Song J. M. (2007). Does the antibacterial activity of
silver nanoparticles depend on the shape of the nanoparticle? A study
of the gram-negative bacterium *Escherichia coli*. Appl. Environ. Microbiol..

[48] Erci F., Cakir-Koc R., Isildak I. (2018). Green synthesis of silver nanoparticles
using Thymbra spicata L. var. spicata (zahter) aqueous leaf extract
and evaluation of their morphology-dependent antibacterial and cytotoxic
activity. Artif. Cells, Nanomed., Biotechnol..

[49] Cheon J. Y., Kim S. J., Rhee Y. H., Kwon O. H., Park W. H. (2019). Shape-dependent
antimicrobial activities of silver nanoparticles. Int. J. Nanomed..

[50] Maillard J. Y., Hartemann P. (2013). Silver as an antimicrobial: facts and gaps in knowledge. Crit Rev. Microbiol.

[51] Gonzalez-Martin, C. Airborne Infectious Microorganisms. Encyclopedia of Microbiology. In Reference Module in Life Sciences; Elsevier, 2019; pp 52–60.

[52] Osman A. I., Abu-Dahrieh J. K., Abdelkader A., Hassan N. M., Laffir F., McLaren M., Rooney D. (2017). Silver-modified η-Al_2_O_3_ catalyst for DME production. J. Phys. Chem.
C.

