# Supplementary material for: Multi-round Recycling of Green Waste for the Production of Silver Nanoparticles: Synthesis, Characterization, and Biological Activity
Source: ACS Omega. 2025 Aug 4;10(32):35793–808. doi: 10.1021/acsomega.5c02607 (PMC12368808; doi:10.1021/acsomega.5c02607)
Supplement: Supplementary file 1 [file ao5c02607_si_001.pdf]

Supporting information

Multi-round recycling of green waste for the production of silver nanoparticles: synthesis, characterization, and biological activity

Andrea Rónavári[1,*], Edi Kachal[1], Nóra Igaz[2], Bettina Szerencsés[3], Bence Kutus[4], Ilona Pfeiffer[3], Mónika Kiricsi[2,#,*], Zoltán Kónya[1,#]

[1] Department of Applied and Environmental Chemistry, Faculty of Science and Informatics, University of Szeged, H-6720, Szeged, Rerrich Béla tér 1., Csongrád-Csanád County, Hungary;

[2] Department of Biochemistry and Molecular Biology, Faculty of Science and Informatics, University of Szeged, H-6726, Szeged, Közép fasor 52., Csongrád-Csanád County, Hungary;

[3] Department of Microbiology, Faculty of Science and Informatics, University of Szeged, H-6726, Szeged, Közép fasor 52., Csongrád-Csanád County, Hungary;

[4] Department of Molecular and Analytical Chemistry, Faculty of Science and Informatics, University of Szeged, H-6720, Szeged, Dóm tér 7-8., Csongrád-Csanád County, Hungary

[#] These authors contributed equally.

* Corresponding authors:

Andrea Rónavári, PhD, Department of Applied and Environmental Chemistry, University of Szeged, Rerrich Béla tér 1. H-6720, Szeged, Hungary, E-mail: ronavari.andrea@gmail.com, ronavari.andrea@szte.hu

Mónika Kiricsi, PhD, Department of Biochemistry and Molecular Biology, University of Szeged, Közép fasor 52. H-6726, Szeged, Hungary. E-mail: kiricsim@gmail.com, kiricsim@bio.u-szeged.hu

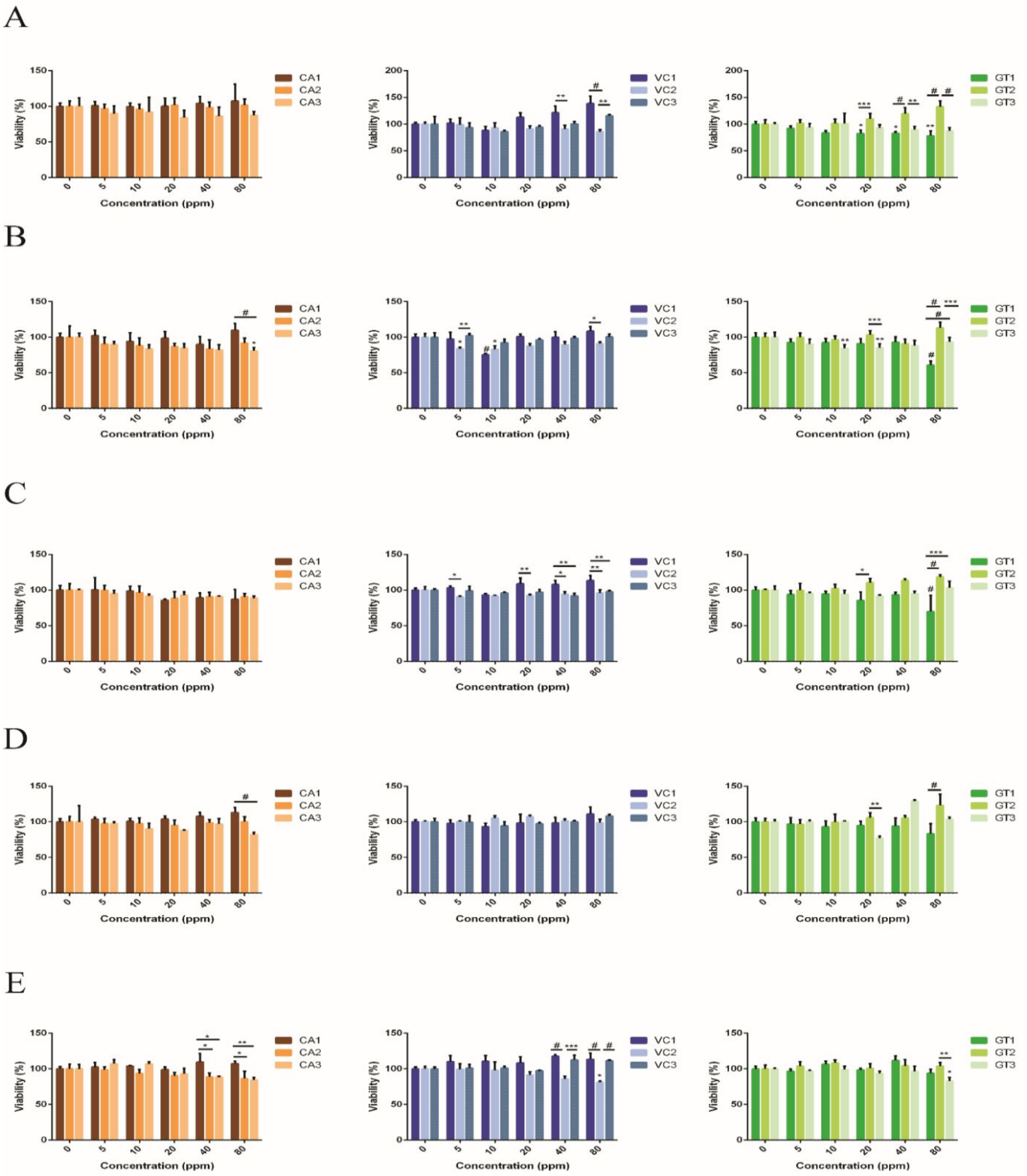

Figure S1. The viability of (A) A549, (B) MCF-7, (C) MCF-7 KCR, (D) MDA-MB-231 cancerous, and (E) MRC-5 non-cancerous cells 24 hours after AgNP-free green waste extract (CA, VC, GT) treatments. Two-way ANOVA, Tukey's multiple comparisons test, *P < 0.05; **P < 0.005; ***P < 0.001; $^{\#}$P < 0.0001

Table S1. Minimal inhibitory concentration of multi-round generated green waste extracts against bacteria (*E. coli* and *B. megaterium*) and fungi (*A. niger* and *C. neoformans*). Plant extracts were achieved by first, second, and third extraction rounds of coffee (CA1-CA3), Virginia creeper (VC1-VC3) and green tea (GT1–GT3) waste.

	Bacillus megaterium SZMC 6031	***Escherichia coli*** SZMC 0582	***Aspergillus niger*** SZMC 0050	***Cryptococcus neoformans*** IFM 5844
CA1	-	-	-	-
CA2	-	-	-	-
CA3	-	-	-	-
VC1	-	-	-	-
VC2	-	-	-	-
VC3	-	-	-	-
GT1	-	-	-	-
GT2	-	-	-	-
GT3	-	-	-	-

-: no growth inhibition was observed

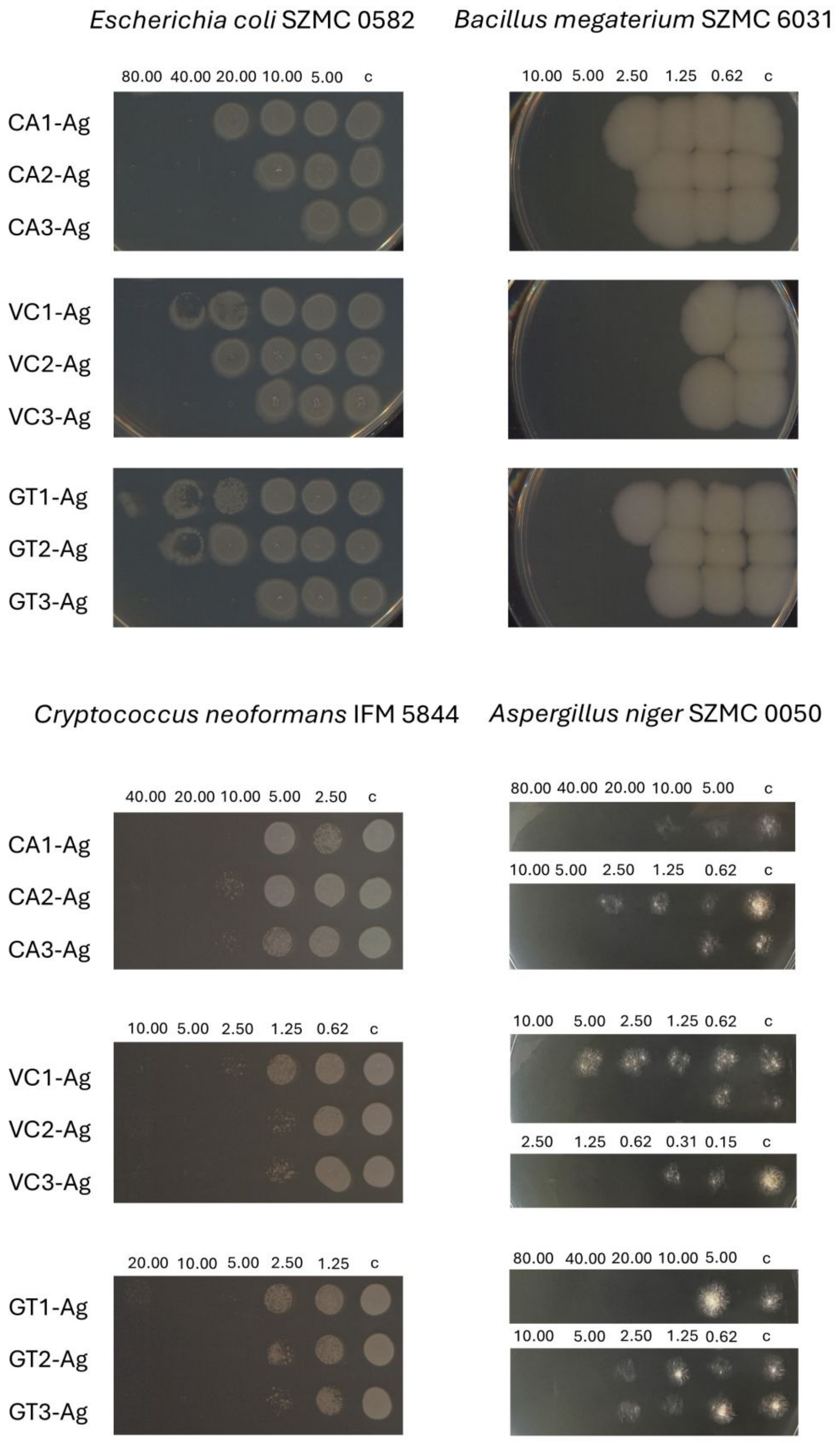

Figure S2. Growth of *E. coli*, *B. megaterium, A. niger* and *C. neoformans* after AgNP-treatment. The letters indicate plant extracts (CA: coffee, GT: green tea, VC: *Virginia creeper*) and the AgNPs produced by using green waste extracts (CA-Ag, VC-Ag, GT-Ag) are depicted at the left side of the picture while the applied concentrations are at the top of the figures.

c: growth control
